# Species-Specific Chromosome Engineering Greatly Improves Fully Human Polyclonal Antibody Production Profile in Cattle

**DOI:** 10.1371/journal.pone.0130699

**Published:** 2015-06-24

**Authors:** Hiroaki Matsushita, Akiko Sano, Hua Wu, Zhongde Wang, Jin-an Jiao, Poothappillai Kasinathan, Eddie J. Sullivan, Yoshimi Kuroiwa

**Affiliations:** 1 SAB Biotherapeutics, Inc., Sioux Falls, South Dakota, United States of America; 2 Kyowa Hakko Kirin, Co., Ltd., Chiyoda-ku, Tokyo, Japan; 3 Department of Animal, Dairy and Veterinary Sciences, Utah State University, Logan, Utah, United States of America; 4 Trans Ova Genetics, Sioux Center, Iowa, United States of America; 5 Hematech, Inc., Sioux Falls, South Dakota, United States of America; Chang Gung University, TAIWAN

## Abstract

Large-scale production of fully human IgG (hIgG) or human polyclonal antibodies (hpAbs) by transgenic animals could be useful for human therapy. However, production level of hpAbs in transgenic animals is generally very low, probably due to the fact that evolutionarily unique interspecies-incompatible genomic sequences between human and non-human host species may impede high production of fully hIgG in the non-human environment. To address this issue, we performed species-specific human artificial chromosome (HAC) engineering and tested these engineered HAC in cattle. Our previous study has demonstrated that site-specific genomic chimerization of pre-B cell receptor/B cell receptor (pre-BCR/BCR) components on HAC vectors significantly improves human IgG expression in cattle where the endogenous bovine immunoglobulin genes were knocked out. In this report, hIgG1 class switch regulatory elements were subjected to site-specific genomic chimerization on HAC vectors to further enhance hIgG expression and improve hIgG subclass distribution in cattle. These species-specific modifications in a chromosome scale resulted in much higher production levels of fully hIgG of up to 15 g/L in sera or plasma, the highest ever reported for a transgenic animal system. Transchromosomic (Tc) cattle containing engineered HAC vectors generated hpAbs with high titers against human-origin antigens following immunization. This study clearly demonstrates that species-specific sequence differences in pre-BCR/BCR components and IgG1 class switch regulatory elements between human and bovine are indeed functionally distinct across the two species, and therefore, are responsible for low production of fully hIgG in our early versions of Tc cattle. The high production levels of fully hIgG with hIgG1 subclass dominancy in a large farm animal species achieved here is an important milestone towards broad therapeutic applications of hpAbs.

## Introduction

Polyclonal antibodies (pAbs) have been used as a therapeutic agent for many years in treating a variety of diseases, particularly for infectious diseases. However, current IgG products, such as human intravenous immunoglobulin (IVIG), monoclonal antibodies, and animal derived polyclonal antibodies, have known limitations. The limitation of human polyclonal antibody products has been the requirement to obtain high volumes of plasma from convalescent human donors with high titers to make commercial products. Although animal-derived pAbs could be an alternative to human polyclonal antibody products, they typically have relatively high toxicity since these animal-derived antibody products are highly immunogenic that can cause a variety of severe adverse effects such as allergic reactions. To avoid serious side effects, animal antibodies are usually processed into smaller F(ab) or F(ab’)_2_ fragments which significantly reduces their half-life and potency, and typically restricts their availability to a limited number of administrations. Disadvantage for monoclonal antibodies is that they are directed against a single epitope that may be subject to rapid mutational escape and the cost of manufacturing mAb products are very high.

Human polyclonal antibodies (hpAbs) produced from transgenic animals could be an important alternative to human plasma-derived IVIG therapy [1–5] with potentially a wider range of applications. However, availability of such transgenic animal species was limited to mice [6] and rabbits, thus, due to their small body size, they were obviously unsuitable for large-scale production of hpAbs. We previously developed a Tc bovine system to produce fully hIgG in plasma and reported the generation of one Tc bovine (calf 468) with a human artificial chromosome (κHAC) vector containing the entire human immunoglobulin heavy (h*IGH*) and κ light chain (h*IGK*) loci, and two bovine *IGH* (b*IGH*) loci, b*IGHM* and b*IGHML1*, being disrupted, designated as κHAC/*IGHM* ^*−/−*^ *IGHML1* ^*−/−*^ [4]. However, the amount of “fully” hIgG (hIgG/hIgκ) was only ~0.3 g/L on average due to the fact that majority of IgG expressed by κHAC/*IGHM* ^*−/−*^ *IGHML1* ^*−/−*^ Tc bovine is chimeric IgG (cIgG, containing human heavy chain and bovine light chain). Apparently, this very low level of fully hIgG is not good enough for a practical use of hpAbs for human therapeutic purpose.

As an effort to overcome these problems, in our recent report [7] we constructed a new HAC vector, cKSL-HACΔ, and transferred this HAC to new double knockout (DKO) *IGHM* ^*−/−*^ *IGHML1* ^*−/−*^ cell lines, which were established through breeding. The resulting cKSL-HACΔ Tc cattle increased total human IgG levels (including fully human IgG and cIgG) drastically, but fully human IgG level was still low, due to the presence of intact bovine light chain gene in this series of Tc calves [7]. In order to address the low fully human IgG issue, we carried out bovine lambda light chain knock out [8], because lambda light chain is the dominant (~95%) light chain in bovine. Using the established triple knockout (TKO) cell line, we generated cKSL-HACΔ/TKO cattle which showed a significant improvement in fully human IgG level in sera or plasma [8].

Despite the significant improvement enhanced by HAC modifications and bovine lambda light chain knock out, we still observed a phenomenon which could be related to potential problems in class-switch recombination in our Tc cattle: high human IgG2/IgG1 subclass ratio and high expression level of trans-class switched bovine IgG (t-bIgG). Regarding human IgG subclass, for the production of therapeutic hpAbs, human IgG1 would be preferred to other IgG subclass in most cases due to longer half-life, higher affinity to Fc receptor (thus greater effector functions associated with IgG1 Fc such as ADCC and CDC) and strong complement fixation, and the majority of therapeutic mAbs in the market is IgG1 [9]. Therefore, for these practical reasons, Tc bovines producing high levels of fully human IgG with IgG1 dominancy would be ideal for many therapeutic applications, particularly in the fields of infectious diseases and cancer. However, Tc cattle we reported previously [8] showed human IgG2 dominancy. In addition, high levels of t-bIgG in our early versions of Tc cattle were also observed [4].

Since immunoglobulin gene organization and function evolved distinctly among species [10], there could be highly species-specific genomic sequences in immunoglobulin-related genes. However, little is known to what extent these species-specific sequence differences are influential to immunoglobulin function across species. Here, we hypothesize that there could be interspecies-incompatible genomic sequences between human and bovine that could functionally hamper high production of fully hIgG in the bovine environment, and we therefore performed species-specific chromosome engineering on our HAC vectors and evaluated each of these new vectors in cattle to test our hypothesis which is based on the concept of “species incompatibility”. Specifically, we successfully constructed a series of new HAC vectors in which the h*IGHG1* gene class switch regulatory element, human I_γ1_-S_γ1_ (hI_γ1_-hS_γ1_) were bovinized (human sequences were replaced with bovine sequences). For one HAC vector, the h*IGHG1* gene transmembrane and cytoplasmic domains were also bovinized. The results from a series of Tc bovine demonstrated that these species-specific modifications at chromosome levels drastically increased fully hIgG production levels, decreased t-bIgG expression, and also improved hIgG subclass distribution, strongly suggesting that these particular species-specific sequence differences between human and bovine are functionally distinct across the species and are the major cause for low production of fully hIgG and abnormal IgG subclass distribution in our previous genotypes of Tc cattle.

## Results

### Concept and strategy of KcHACΔ vector construction

In a series of our study, we hypothesized that there could be some interspecies-incompatibilities between human and bovine that may generally hamper high production of fully hIgG in the bovine environment. In a previous report, we firstly addressed the IgM-based pre-BCR/BCR function issue by a strategy aiming at resolving species incompatibilities between bovine Ig-α/Ig-β and human IgM heavy chain, and between bovine surrogate light chain (SLC) and human IgM heavy chain [7]. In that strategy, we replaced part of human IgM heavy chain constant region (CH2 to TM2) with the corresponding sequences of bovine IgM, and also we included human SLC loci along with human lambda light chain locus in the HAC vector, resulting in a cKSL-HACΔ [7]. In the current study, the same strategy was employed to κHAC [4] to construct KcHACΔ vector without implementing human SLC.

**Fig 1:**
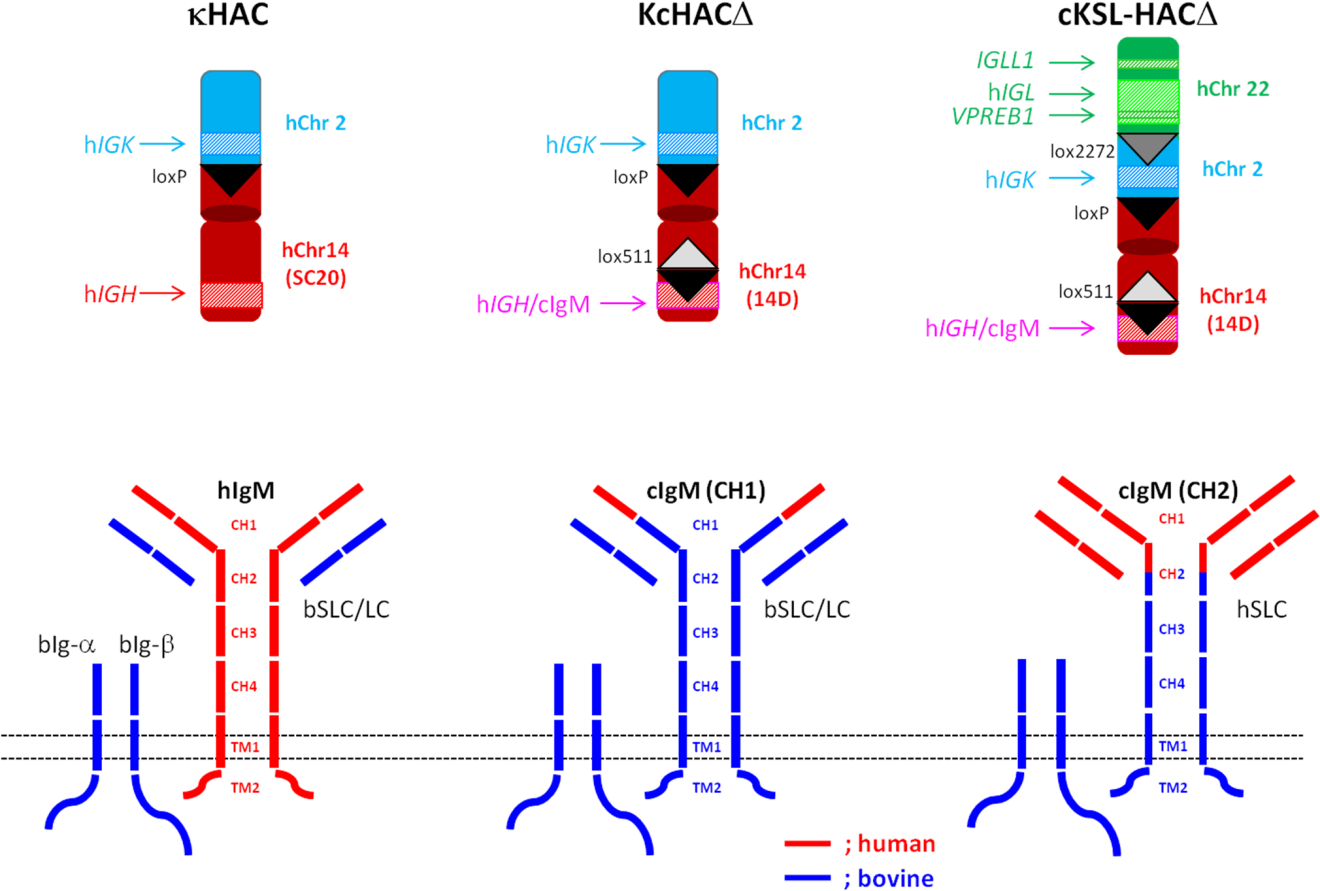
Comparison of IgM in κHAC, KcHACΔ and cKSL-HACΔ vectors. Overall structure of the κHAC, KcHACΔ and cKSL-HACΔ vectors were illustrated at the upper paneland their IgM structures were illustrated at the lower panel. The KcHACΔ vector is a derivative of the original κHAC where part of the h*IGHM* gene constant region, the CH1 through TM domains, is bovinized with the bovine-origin sequence. Because of this modification, the KcHACΔ vector expresses the cIgM(CH1) protein on pre-B/B cell surface. The previously reported cKSL-HACΔ vector is composed of three different hChr fragments, hChr14 (14D), hChr2 and hChr22 containing the entire h*IGL* and surrogate light chain (h*VPREB1* and h*IGLL1*) loci. In this vector, part of the h*IGHM* gene constant region, the CH2 through TM domains, is bovinized to express the cIgM(CH2) protein. SLC, surrogate light chain; LC, light chain.

To construct KcHACΔ vector, part of the h*IGHM* constant region gene (CH1 through TM2 domains) was bovinized so that in such a chimeric IgM structure [which was named as cIgM(CH1) for chimeric human IgM bovinized at CH1 through TM2 domain], the pre-BCR/BCR could interact more effectively with bovine surrogate light chain, orthodox light chain and Ig-α/Ig-β molecules for better pre-BCR/BCR signaling (Fig 1). On the other hand, in the previously reported cKSL-HACΔ vector [7], part of the h*IGHM* constant region gene (CH2 through TM2 domains) was differently bovinized and, furthermore, the human surrogate light chain h*VPREB1* and h*IGLL1* (λ5 in the mouse) genes were introduced with the hChr22 fragment so that in such a chimeric IgM structure, which was named as cIgM(CH2) for chimeric human IgM bovinized at CH2 through TM2 domain, pre-BCR/BCR could pair with human SLC and could also interact with bovine Ig-α/Ig-β molecules for better pre-BCR/BCR signaling (Fig 1). Due to the species-specific distinct chimeric sequences of hIgM, cIgM(CH1) and cIgM(CH2) proteins, pre-BCR/BCR function/signaling in each HAC vector (i.e. κHAC, KcHACΔ, cKSL-HACΔ, respectively, in Fig 1) could affect B cell developmental fate, and eventually hIgG production profile, differently. Likewise, we also constructed the KcHAC vector where the hChr2 fragment (κTL1) [7] was translocated to the SC20 fragment bearing the bovinized IgM, cIgM(CH1), sequence.

### Construction of KcHACΔ vector in chicken DT40 cells

**Fig 2:**
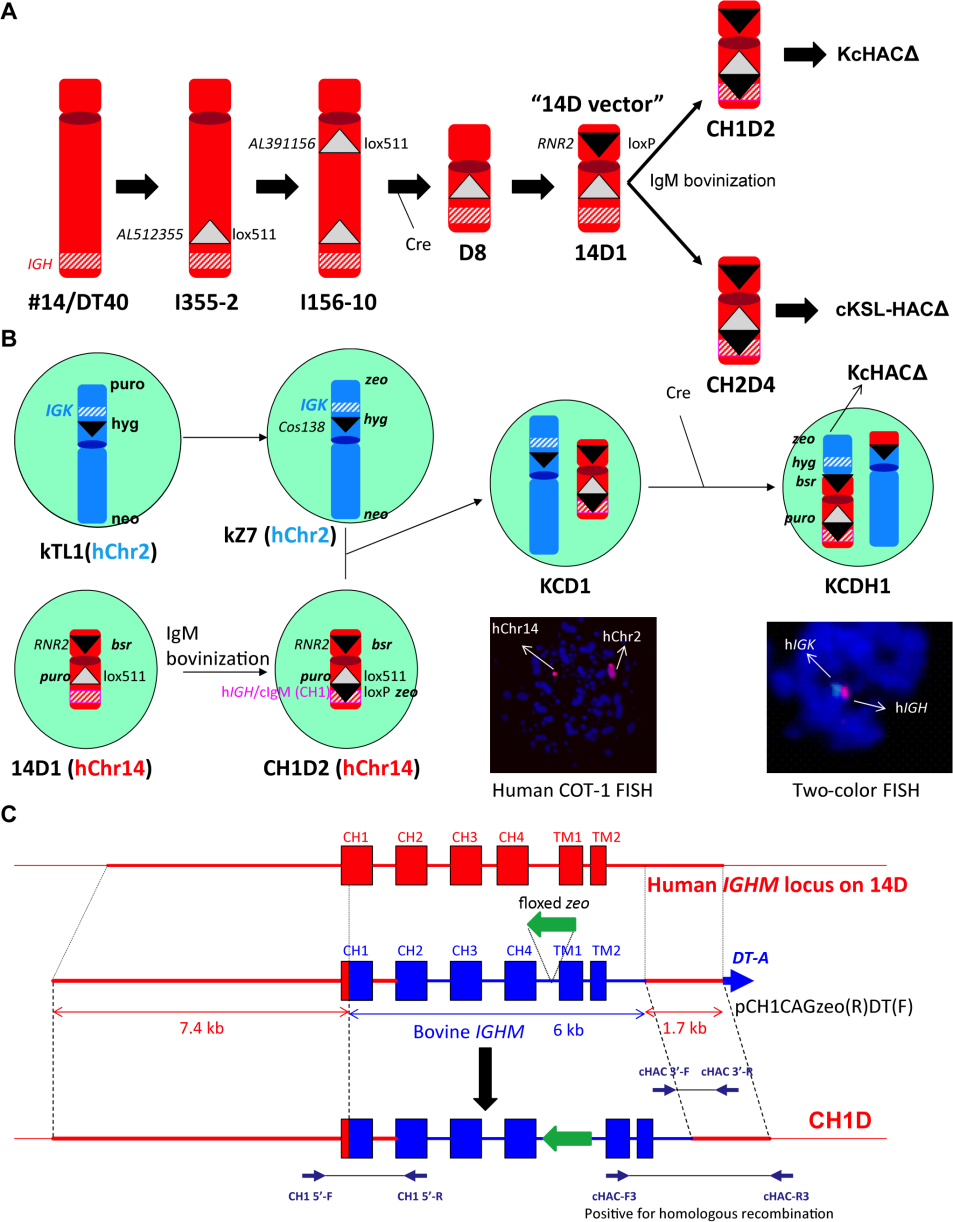
Construction of the KcHACΔ. (A) Construction of CH1D vector. 14D1 was constructed as previously described [7]. Subsequently, the cIgM(CH1) or cIgM(CH2) bovinization generated CH1D2 or CH2D4, which was used for the KcHACΔ or cKSL-HACΔ vector construction [7], respectively. (B) Construction of the KcHACΔ vector in chicken DT40 cells. (C) cIgM(CH1) modification for the CH1D construction. The bovinization vector pCH1CAGzeo(R)DT(F) comprises 7.4 kb and 1.7 kb human genomic DNA as a long and short arm, respectively, 6 kb of the bovine *IGHM* constant region genomic DNA covering the CH1 through TM2 domains where the floxed, CAG promoter-driven *zeo* gene cassette was integrated between the CH4 and TM1 intron, and the *DT-A* gene. After the homologous recombination, part of the h*IGHM* constant region, the CH1 through TM2 domains, was bovinized on the CH1D.

**Fig 3:**
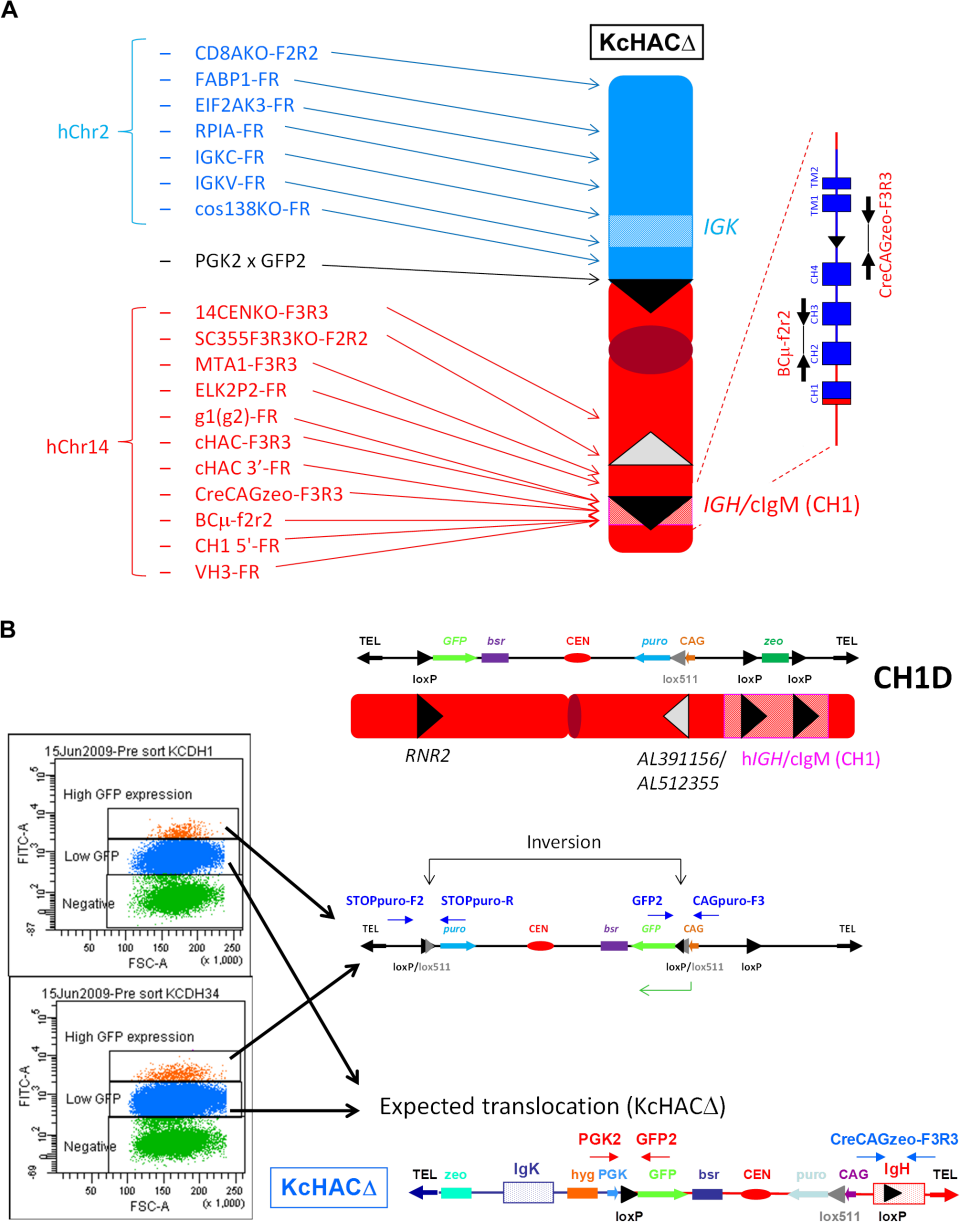
PCR genotyping for KcHACΔ construction. (A) Extensive genomic PCR for genotyping of the KcHACΔ vector. Location of each genomic PCR primer pair is depicted in relation to the KcHACΔ vector structure. (B) Occurrence of the chromosome inversion on the CH1D. A recombination between the *loxP* at the *RNR2* locus and the *lox511* at the deletion junction site *AP391156*/*AP512355* caused the inversion that also reconstituted the CAG promoter-driven *GFP* gene, leading to the higher GFP expression than the PGK promoter-driven *GFP* gene from the cKSL-HACΔ vector. The inversion was confirmed by genomic PCR, STOPpuro-F2R2 and GFP2 x CAGpuro-F3.

The KcHACΔ vector was constructed in DT40 hybrid cells as outlined in Fig 2A and 2B, using the chromosome cloning system. Human chromosome 2 derived DT40 clone κTL1 [7] was transfected with targeting vector pTELCAGzeoCD8A to replace puromycin resistance gene with zeocin resistant gene. One hundred of zeocin resistant colonies were examined for puromycin sensitivity and 31 puromycin sensitive colonies were screened by PCR with primers Puro-FR for the detection of remained puromycin cassette and CD8AKO-FR, F2R2 for the detection of homologous recombination. Seven puro-FR negative and CD8AKO-FR, F2R2 positive clones were further screened by PCR with primers FABP1-FR, EIF2AK3-FR, RPIA-FR, IGKC-FR, IGKV-FR and cos138KO-FR. Five clones were further examined by FISH and kZ7 and kZ20 were selected for further steps. Human chromosome 14 derived HAC basal vector “14D” DT40 clone [7] 14D1 was transfected with a targeting vector pCH1CAGzeo(R)DT(F) for bovinization of part of human IgM constant region (Fig 2C). Twenty-six zeocin resistant colonies were screened with PCR cHAC-FR for homologous recombinant and PCR CH3F3xCH4R2 for non-targeted cells. 10 positive clones were subjected to extensive PCR screening with 11 sets of primers (S1A Fig). Selected 4 clones were examined by FISH (S1B Fig). Positive clones CH1D2 and CH1D10 were fused with chromosome 2 containing DT40 clones kZ7 and kZ20 (whole cell fusion). Twelve hygromycin and blasticidin resistant colonies were subjected to PCR screening with IGKV-FR for chromosome 2/*IGK* locus and g1(g2)-FR for chromosome 14/*IGH* locus. All 12 clones were positive for both PCR and further screened with additional 6 PCR for the presence of kZ and additional 12 PCR for the presence of CH1D (Fig 3A and S2A Fig). Correct hybrid clone KCD1 and KCD34 were further confirmed by FISH and selected for further steps (S2B Fig). In order to translocate *IGK* locus containing chromosome 2 fragment onto cIgM(CH1) modified 14D vector (CH1D) at *RNR2* locus, Cre expression vector was transfected to the above KCD1 and KCD34 clones to initiate Cre-*loxP* recombination between *loxP* at *cos138* locus on kZ (truncated human chromosome 2) and *loxP* at *RNR2* locus on CH1D (Fig 2B). Recombinants were enriched by sorting GFP positive cells as GFP expression is conferred by reconstitution of the PGK promoter-*loxP*-*GFP* cassette at the translocation site. Sorting was conducted three times which resulted in two distinct GFP positive populations with different expression levels (Fig 3B). The lower GFP population contained the successfully translocated to generate KcHACΔ vector, which was determined by PCR primers; PGK2 x GFP2 confirmed the junction between chromosome 14 fragment and chromosome 2 fragment generated by chromosome translocation, and PCR primers, CreCAGzeo-F3R3, confirmed the successful removal of CAG promoter-*zeo* cassette in the cIgM(CH1) site (Fig 3B, S3A Fig). The higher GFP population contained the inverted CH1D fragment between the *loxP* at the *RNR2* locus and the lox511 at the locus *AL512355*/*AL391156* by a Cre-mediated recombination which was confirmed by PCR primers, CAGpuro-F3 x GFP2 and STOPpuro-F2 x STOPpuro-R, followed by direct sequencing (Fig 3B). KCDH1(3L) and KCDH34(3L) was finally identified as DT40 hybrid cell lines retaining the KcHACΔ based on the result of extensive genomic PCR (Fig 3A, S3B Fig) and two color-FISH (Fig 2B). The KcHAC vector was similarly constructed in DT40 cells. The hChr2 fragment (κTL1) was translocated to the SC20 fragment [11] bearing the bovinized cIgM(CH1).

### Concept and strategy for construction of of isHAC, istHAC and isKcHACΔ vectors

Our second strategy was to address whether IgG class switch regulatory element is also involved in species-specific regulation. Class switch recombination that determines IgH isotype is preceded by transcription from each *IGH* locus-associated switch region (S_H_), called germline transcript [12]. Each *IGH* constant region (C_H_) gene is linked with its own S_H_ region which is also associated with its own I_H_ exons. The germline transcript, I_H_-S_H_-C_H_ (eventually spliced to mature I_H_-C_H_), is driven by the promoter/enhancer elements located just 5’ of the I_H_ exons and those elements are cytokine or other activator-responsive. In a simple model of class switch, the specific activators and/or cytokines induce the germline transcript from its activator/cytokine-responsive I_H_ promoter/enhancer. The 3’E_α_ element further enhances the transcription of the I_H_-S_H_-C_H_ sequence. This transcription causes the switch region to be relaxed so that it can be targeted by the enzyme, activation-induced cytidine deaminase (AID), which causes fusion with another S_H_ region, leading to class switch. Our hypothesis was that, for example, the hI_γ1_-hS_γ1_ regulatory element linked with the h*IGHG1* gene is somehow incompatible with such bovine activators/cytokines-induced DNA-binding proteins to efficiently induce class switch to hIgG1, due to the species-specific sequence differences (S4*A* and S4*B* Fig). This may be why many early versions of Tc bovines showed hIgG2-dominancy instead of hIgG1 that is a major subclass in humans.

**Fig 4:**
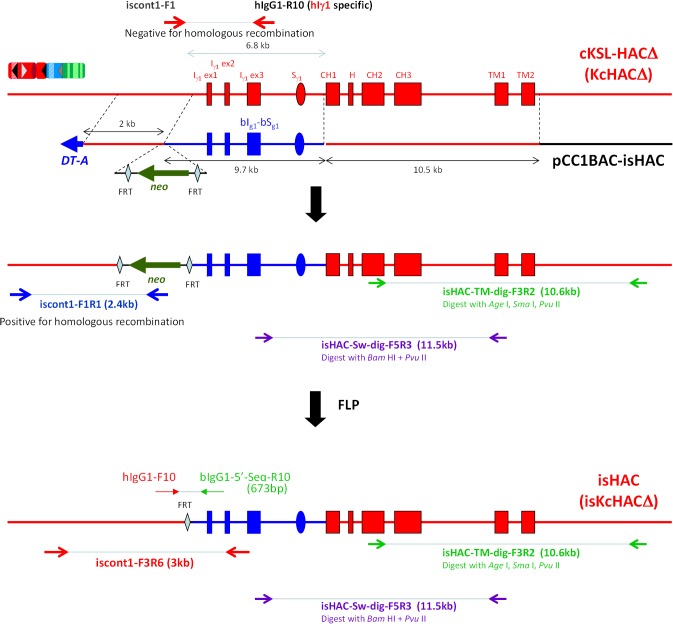
Construction of the isHAC and isKcHACΔ vectors. A flow of the isHAC and isKcHACΔ vector construction are illustrated. The bovinizing vector pCC1BAC-isHAC is a BAC-based one (backbone is pCC1BAC vector), consisting of 10.5 kb and 2 kb of genomic DNA as a long and short arm, respectively, 9.7 kb of the bovine genomic DNA covering the bovine I_γ1_-S_γ1_ and its surrounding region to replace the human corresponding 6.8 kb of I_γ1_-S_γ1_ region, the chicken β-actin promoter-driven *neo* gene flanked by *FRT* sequence and the *DT-A* gene. After the targeted bovinization, the *neo* cassette is removed by FLP introduction.

**Fig 5:**
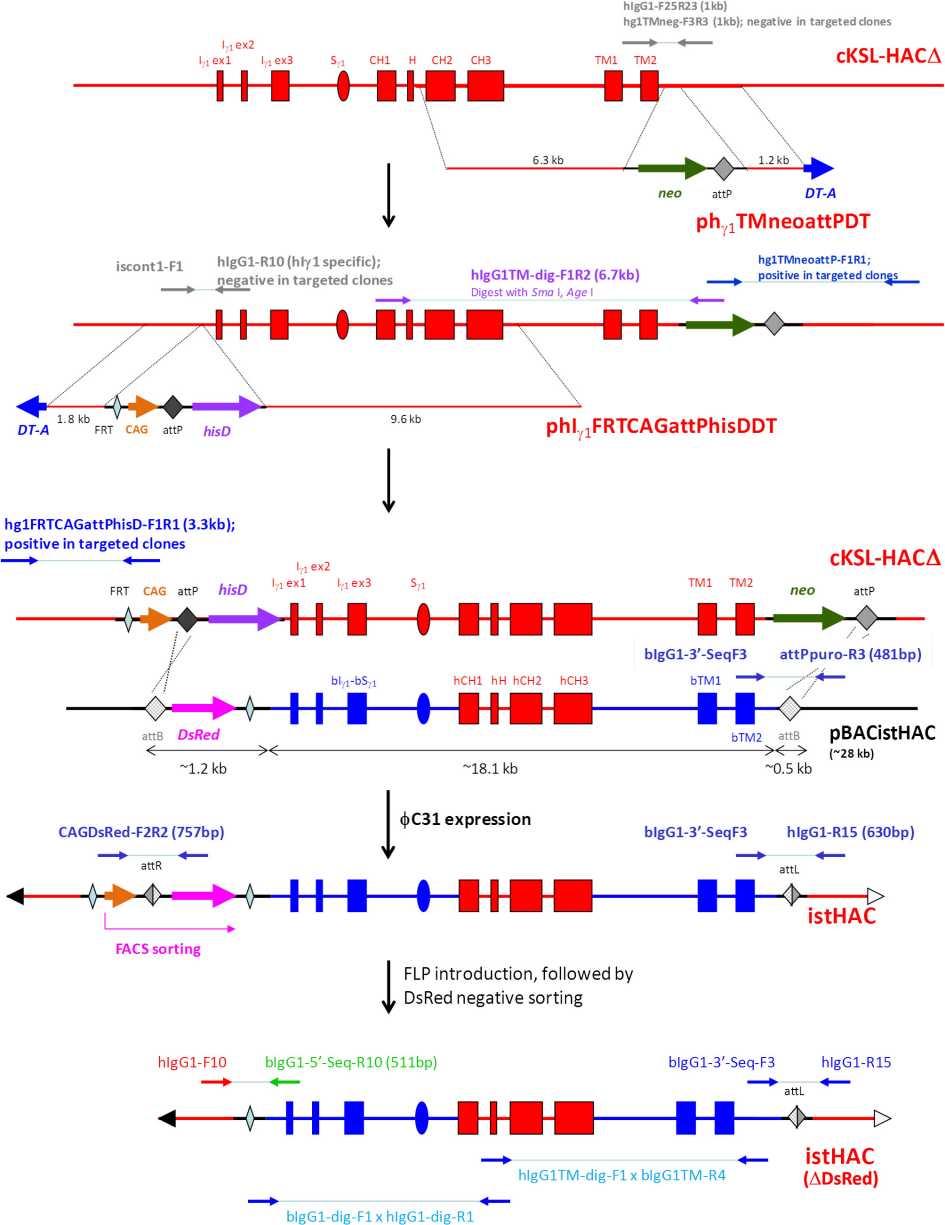
Construction of the istHAC vector. A flow of the istHAC vector construction is illustrated. The *attP* sequence is integrated at 5’ side of the hI_γ1_ exon 1 and 3’ side of the h*IGHG1* TM2 by the targeting vectors phI_γ1_FRTCAGattPhisDDT and ph_γ1_TMNeoattPDT, respectively. Then, the replacement vector pBAC-istHAC is introduced with the ΦC31 recombinase to bring about the *attP*/*attB* recombination to replace the flanked region. The successful replacement causes the CAG promoter-driven DsRed gene to be reconstituted to provide red fluorescence for sorting. Finally, the DsRed cassette is removed by the FLP expression.

Based on the above hypothesis, we bovinized the hI_γ1_-hS_γ1_ class switch regulatory element with that of the b*IGHG1* gene to construct the isHAC vector containing the bI_γ1_-bS_γ1_ sequence upstream of the hC_γ1_ region on the cKSL-HACΔ vector (Fig 4). Moreover, the transmembrane/cytoplasmic domains of the h*IGHG1* gene on the isHAC vector were further bovinized with sequences of the b*IGHG1* gene transmembrane/cytoplasmic domain to generate the istHAC (Fig 5), considering the species-specific sequence differences (S4B Fig). Since the cKSL-HACΔ and KcHACΔ vectors might potentially have functional differences, as seen in the DKO background, and it was uncertain how differently these two HAC vectors would behave in the TKO background lacking the b*IGL* expression, we decided to also bovinize the KcHACΔ vector to generate the isKcHACΔ vector containing the bI_γ1_-bS_γ1_ sequence upstream of the hC_γ1_ region on the KcHACΔ (Fig 4).

### Construction of the isHAC and isKcHACΔ vectors

**Fig 6:**
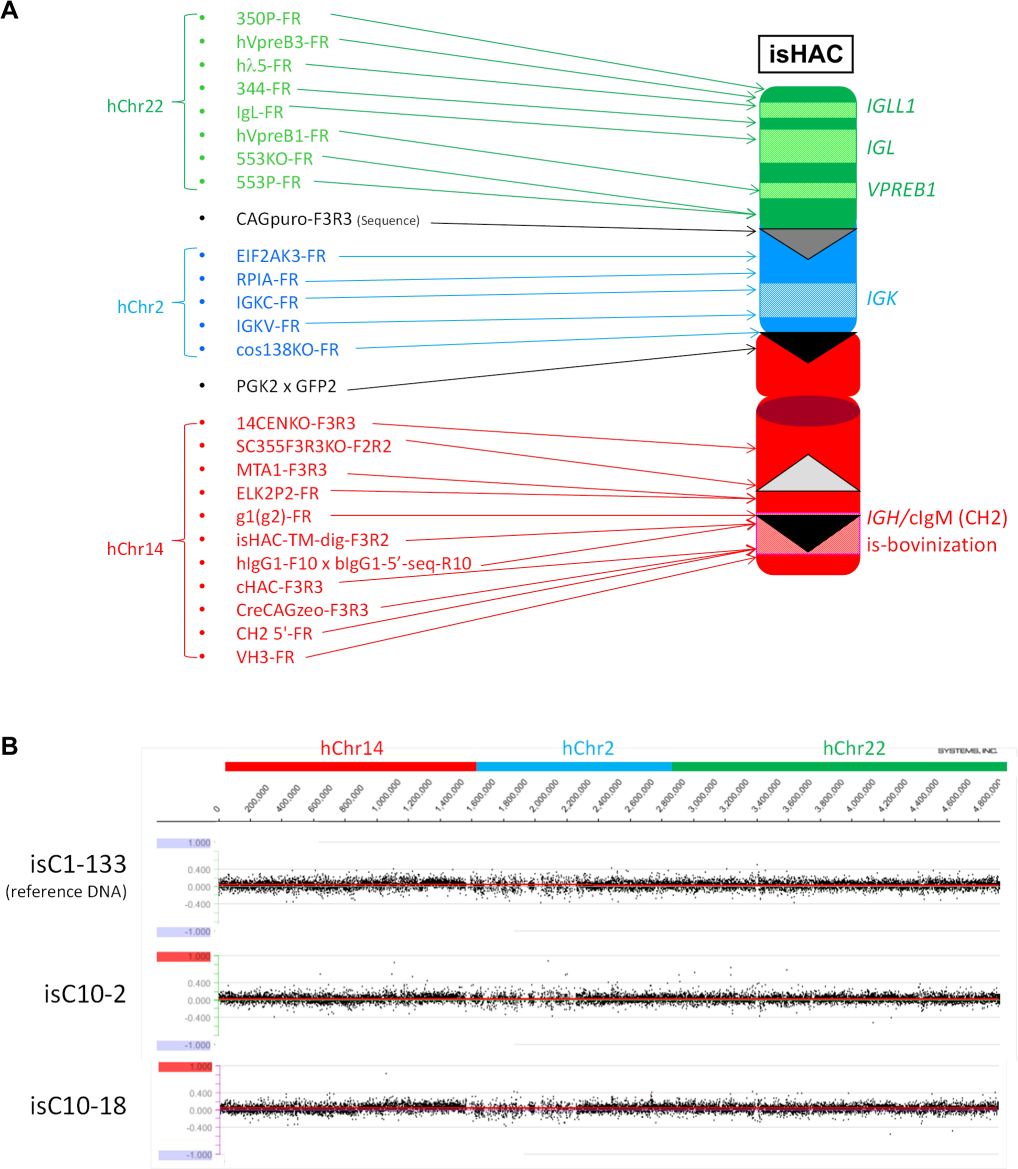
Analysis of constructed isHAC vector. (*A*) Extensive genomic PCR for genotyping of the isHAC vector. Location of each genomic PCR primer pair is depicted in relation to the isHAC vector structure. (*B*) CGH analysis among three different CHO clones containing the isHAC vector. DNA from isC1-133 was used as a reference. There was no apparent structural difference in the isHAC among the three cell lines.

Outline of the isHAC and isKcHACΔ vector construction is depicted in Fig 4. The targeting vector pCC1BAC-isHAC was constructed (S5 Fig) and used to bovinize the I_γ1_-S_γ1_ region on the cKSL-HACΔ or KcHACΔ. Clones cKSLDD1 and cKSLDD10, chicken DT40 cell lines retaining the cKSL-HACΔ obtained by MMCT from cKSLDH22 (2L) [7], were electroporated with the targeting vector pCC1BAC-isHAC. Sixty-seven and 47 G418 resistant colonies, derived from cKSLDD1 and cKSLDD10, respectively, were selected and their genomic DNA was subjected to PCR screening with primers, iscont1-F1R1, to identify the occurrence of the homologous recombination and iscont1-F1 x hIgG1-R10 for the detection of non-targeted cells (Fig 4). Twenty-nine and 17 colonies, derived from cKSLDD1 and cKSLDD10, respectively, were positive for homologous recombination, and 11 and 10 colonies, derived from cKSLDD1 and cKSLDD10, respectively, were selected from them. Furthermore, additional 26 diagnostic PCRs were also performed to check structural integrity (data not shown). Clones is1-11 and is10-9 were selected for the subsequent *neo* cassette deletion by introduction of the FLP-expression plasmid. Clone is10-9 was transfected with the FLP-expressing plasmid. Twenty-one hisD resistant colonies were examined by G418-sensitibity and 20 sensitive colonies were obtained, where the *neo* cassette was deleted by the FLP-*FRT* recombination. They were tested for the genomic PCRs with primer iscont1-F3R6 for cells with *neo* cassette deletion and iscont1-F3R3 for cells without *neo* cassette deletion (Fig 4). For selected 5 clones, further 26 diagnostic PCRs were performed. Finally, we selected clone isH9-3, and then it was transferred to CHO cells to establish master cell banks isC10-2 and isC10-18, for which the extensive genomic PCR and CGH were performed to check structural integrity (Fig 6A and 6B). Clone is1-11, which is equivalent to is10-9, was co-transfected with the FLP-expressing plasmid and the DsRed-expressing plasmid. DsRed-positive cells were sorted and subjected to single colony isolation. Eighteen G418-sensitive colonies, where the *neo* cassette was deleted by the FLP-*FRT* recombination, were tested for the genomic PCRs with primer iscont1-F3R6 for cells with *neo* cassette deletion and iscont1-F3R3 for cells without *neo* cassette deletion (Fig 4). For selected 5 clones, further 27 diagnostic PCRs were performed. Finally, we selected clone isH11-S2, and it was transferred to CHO cells to establish master cell bank isC1-133, for which the extensive genomic PCR and CGH were performed to check structural integrity (Fig 6A and 6B).

**Fig 7:**
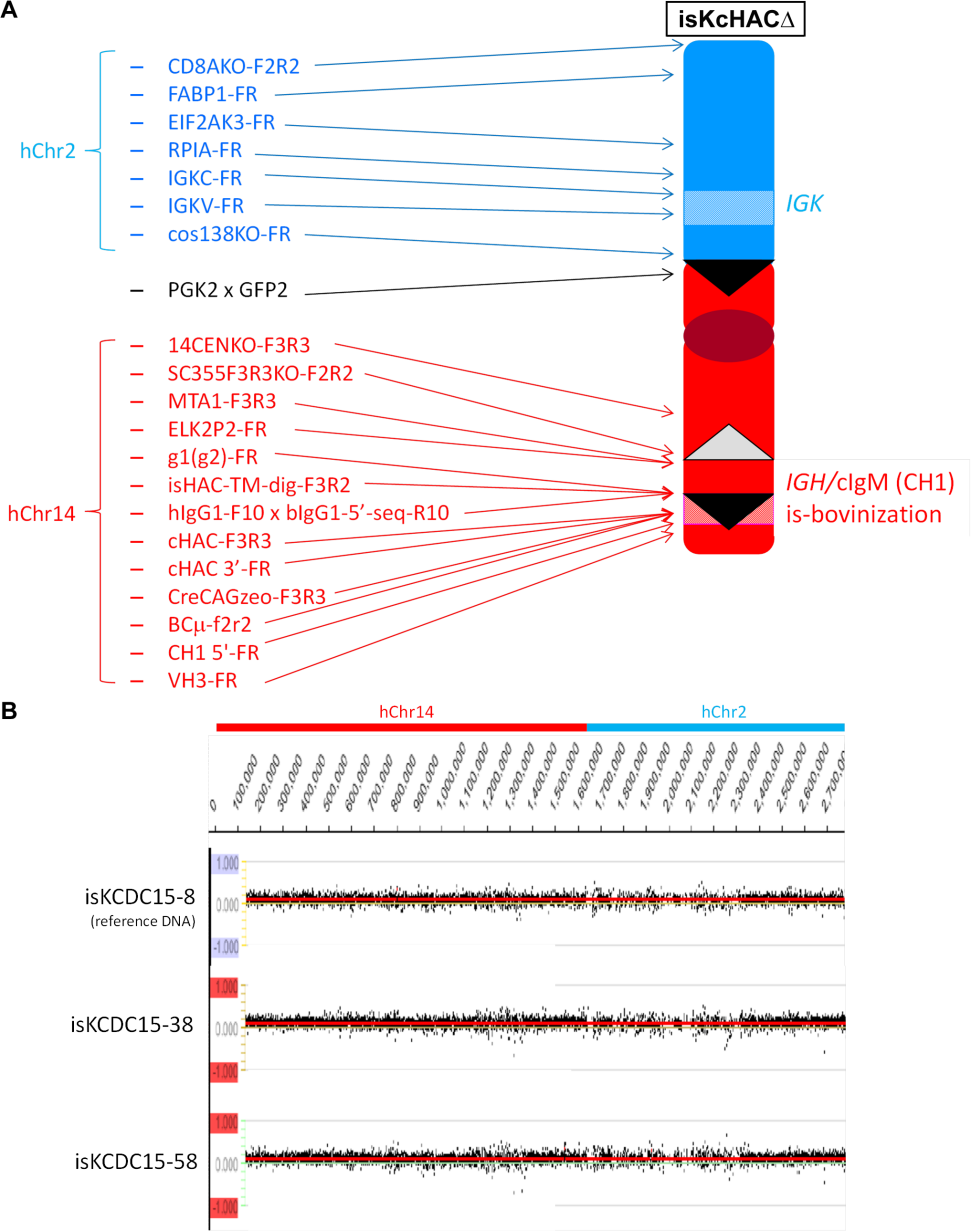
Analysis of constructed isKcHACΔ vector. (*A*) Extensive genomic PCR for genotyping of the isKcHACΔ vector. Location of each genomic PCR primer pair is depicted in relation to the isKcHACΔ vector structure. (*B*) CGH analysis among three different CHO clones containing the isKcHACΔ vector. DNA from isKCDC15-8 was used as a reference. There was no apparent structural difference in the isKcHACΔ among the three cell lines.

The isKcHACΔ was constructed in DT40 cells, similarly to the isHAC. Clones KCDD1-13 and KCDD15-3, chicken DT40 cell lines retaining the KcHACΔ obtained by MMCT from KCDC1 and KCDC15 respectively, were electroporated with the targeting vector pCC1BAC-isHAC. Forty-seven G418 resistant colonies, each derived from KCDD1-13 and KCDD15-3, were selected and their genomic DNA was subjected to PCR screening with primers, iscont1-F1R1, to identify the occurrence of the homologous recombination and iscont1-F1xhIgG1-R10 for the detection of non-targeted cells. Eighteen and 21 colonies derived from KCDD1-13 and KCDD15-3, respectively, were positive for homologous recombination and 8 colonies each were selected from them. Furthermore, additional 25 diagnostic PCRs were also performed to check structural integrity (data not shown). Clones isKCD1-1 and isKCD1-16 derived from KCDD1-13, and clones isKCD15-17 and isKCD15-30 derived from KCDD15-3, were selected for the subsequent *neo* cassette deletion by introduction of the FLP-expression plasmid. Clones isKCD1-1 and isKCD1-16 derived from KCDD1-13, and clones isKCD15-17 and isKCD15-30 derived from KCDD15-3, were co-transfected with the FLP-expressing plasmid and the DsRed-expressing plasmid. DsRed-positive cells were sorted with high purity and pooled cells were tested for the genomic PCRs with primer iscont1-F3R6 for cells with *neo* cassette deletion and iscont1-F3R3 for cells without *neo* cassette deletion (Fig 4). For each sorted pooled cells, further 22 diagnostic PCRs were performed. Finally, these 4 pooled, sorted cells, isKCDH1, isKCDH16, isKCDH17 and isKCDH30 derived from isKCD1-1, isKCD1-16, isKCD15-17 and isKCD15-30, respectively, were confirmed by FISH. They were transferred to CHO cells to establish master cell banks, isKCDC1-22, isKCDC15-8 and isKCDC15-38, derived from isKCD1-16, isKCD15-17 and isKCD15-30, respectively, for which the extensive genomic PCR and CGH were performed to check structural integrity (Fig 7).

### Construction of the istHAC vector in chicken DT40 cells

A scheme of construction of the istHAC vector is depicted in Fig 5. Clone cKSLDD1 was sequentially targeted with two targeting vectors ph_γ1_TMNeoattPDT and phI_γ1_FRTCAGattPhisDDT (Fig 5) to integrate the *attP* sequence at 3’ side of the h*IGHG1* TM2 domain and at 5’ side of the human I_γ1_ region, respectively, which generated two clones ist1-5 and ist1-21. They were co-electroporated with the pBAC-istHAC and ΦC31-expression vectors together to bring about the big DNA replacement. As shown in Fig 5, the expected recombination between *attP* and *attB* should result in reconstitution of the CAG promoter-DsRed gene expression, which can be detected by flow cytometry. DsRed-positive cells were therefore sorted. This sorting process was repeated 2–3 times until purity of DsRed-positive cells reached >95%. Then, cells were subjected to single colony isolation and examined by three diagnostic genomic PCRs, CAGDsRed-F2R2 (positive), bIgG1-3’-SeqF3 x hIgG1-R15 (positive) and bIgG1-3’-SeqF3 x attPPuro-R3 (negative) (Fig 5). As a result, istH5-S16 from ist1-5 and istH21H-S10 from ist1-21 were selected.

**Fig 8:**
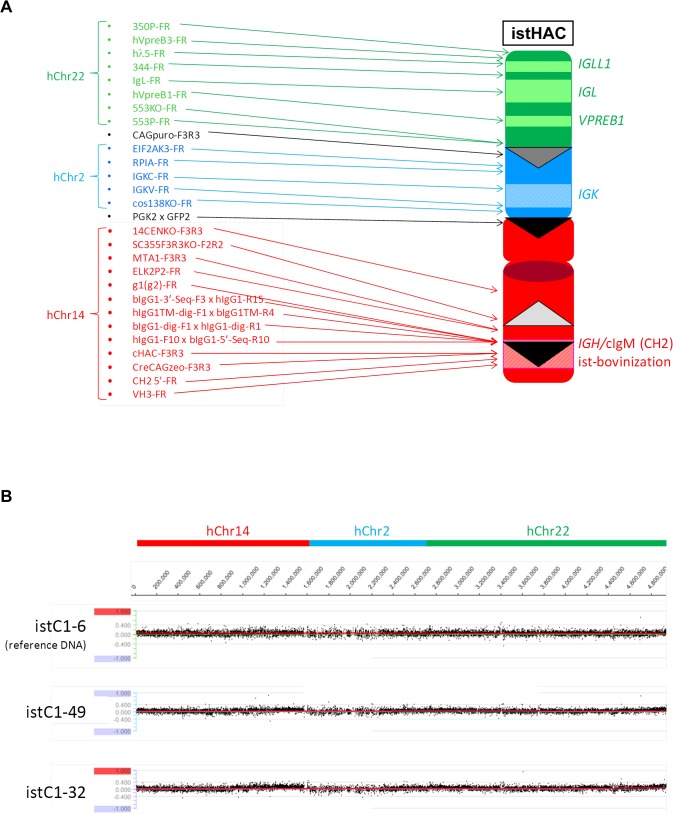
Analysis of constructed istHAC vector. (*A*) Extensive genomic PCR for genotyping of the istHAC vector. Location of each genomic PCR primer pair is depicted in relation to the istHAC vector structure. (*B*) CGH analysis among three different CHO clones containing the istHAC vector. DNA from istC1-6 was used as a reference. There was no apparent structural difference in the istHAC among the three cell lines.

The two selected clones, istH5-S16 and istH21H-S10, were finally transfected with the FLP-expression vector. As shown in Fig 5, FLP expression should cause the removal of the CAG-DsRed gene expression, which can be detected by flow cytometry. DsRed-negative cells were thus sorted, resulting in >95% purity of DsRed-negative cells. Then, cells were subjected to single colony isolation and examined by three diagnostic genomic PCRs, CAGDsRed-F2R2 (negative), bIgG1-3’-SeqF3 x hIgG1-R15 (positive) and hIgG1-F10 x bIgG1-5’-Seq-R10 (positive)(Fig 5). Consequently, istHD16L from istH5-S16 and istHD10L from istH21H-S10 were selected and then were transferred to CHO cells to establish master cell banks, istC1-49 and istC1-6, respectively, for which the extensive genomic PCR and CGH were performed to check structural integrity (Fig 8).

### Human IgG production profile in a series of HAC/*IGHM* ^*−/−*^ *IGHML1* ^*−/−*^ *IGL* ^*−/−*^ (TKO) cattle

**Fig 9:**
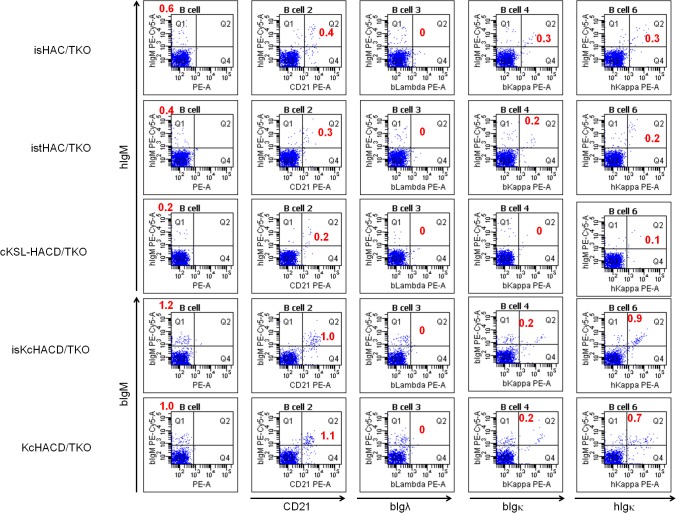
Characterization of isHAC/TKO, istHAC/TKO and isKcHACΔ/TKO cattle by PBMC expression profile. Representative flow cytometry analysis of PBMCs from a series of HAC/TKO calves at newborn stage. For IgM detection, anti-hIgM or anti-bIgM antibody was used. From left to right panels, PBMCs were stained for IgM alone, IgM/bCD21, IgM/bIgλ, IgM/bIgκ and IgM/hIgκ. Each red number represents percentages of cells in Q1 (IgM alone) or Q2 (IgM/bCD21, IgM/bIgλ, IgM/bIgκ and IgM/hIgκ).

**Fig 10:**
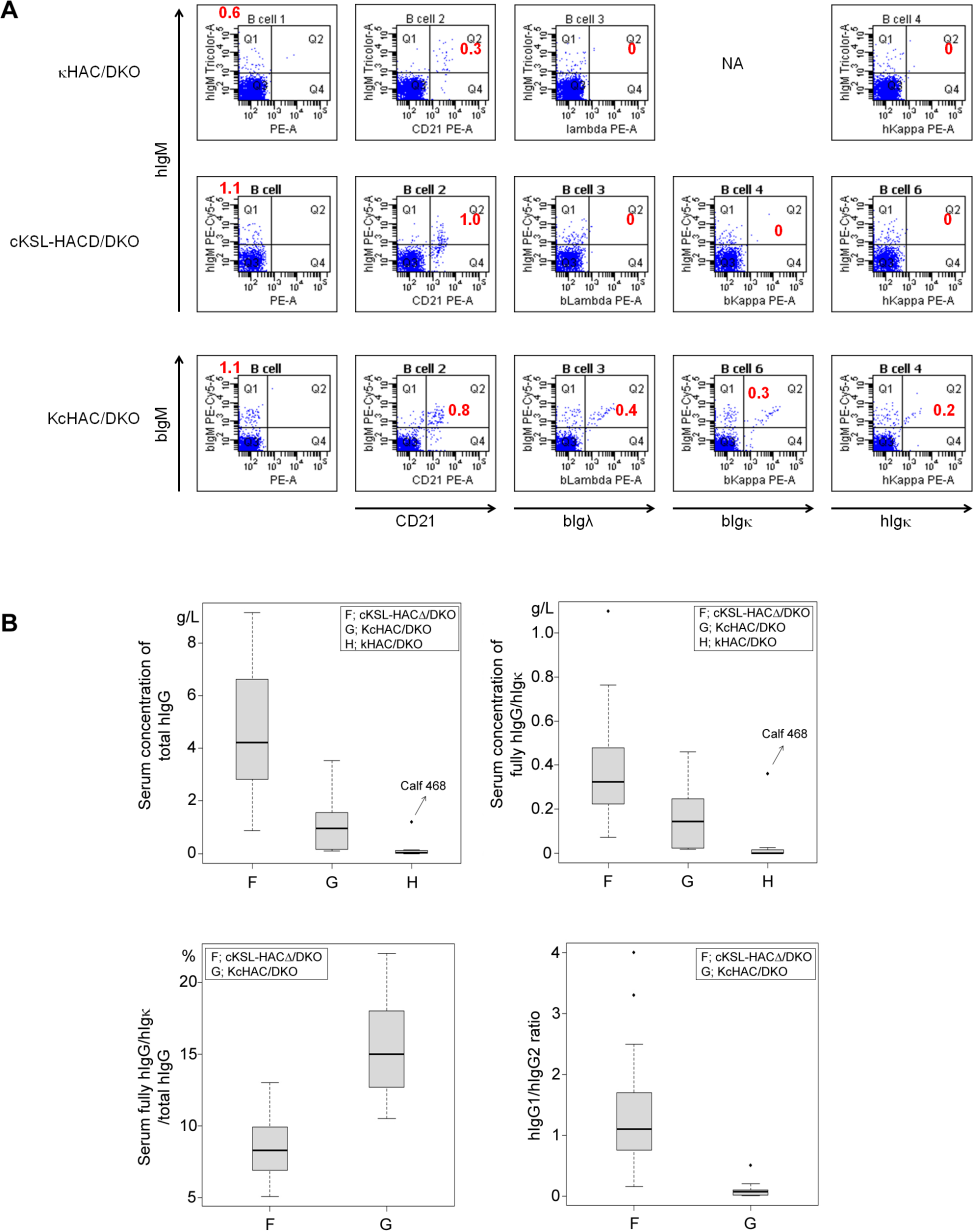
Characterization of κHAC/DKO, cKSL-HACΔ/DKO and KcHAC/DKO cattle by PBMC expression profile and serum IgG profile. (*A*) Representative flow cytometry analysis of PBMCs from a series of HAC/DKO calves at newborn stage. For IgM detection, anti-hIgM or anti-bIgM antibody was used. From left to right panels, PBMCs were stained for IgM alone, IgM/bCD21, IgM/bIgλ, IgM/bIgκ and IgM/hIgκ. Each red number represents percentages of cells in Q1 (IgM alone) or Q2 (IgM/bCD21, IgM/bIgλ, IgM/bIgκ and IgM/hIgκ). NA: not applicable (because, at that time, the anti-bIgκ antibody was not available). (*B*) Box-whisker plots of serum concentrations of total hIgG (g/L)(top left), fully hIgG/hIgκ (g/L)(top right), serum fully hIgG/hIgκ (%)/total hIgG (bottom right) and hIgG1/hIgG2 ratio (bottom left) in a series of HAC/DKO cattle at 5–6 months of age. F, cKSL-HACΔ/DKO (n = 33); G, KcHAC/DKO (n = 12); H, κHAC/DKO (n = 8). Dots represent outliers. The value of calf 468 were indicated with arrows.

The isHAC, istHAC, isKcHACΔ and KcHACΔ vectors were transferred from the CHO master cell banks to the *IGHM* ^*−/−*^ *IGHML1* ^*−/−*^ *IGL* ^*−/−*^ (TKO) bovine cell line by MMCT to generate a series of HAC/TKO calves. Calving efficiency at 270 days of gestation was around 7% out of recipients implanted (Table 1). In order to address the impact of the ablation of the b*IGL* expression on B cell development, we performed flow cytometry on all four new genotypes of HAC/TKO calves at newborn stage and compared with that of cKSL-HACΔ (Fig 9). In comparison with the DKO background (Fig 10), percentages of hIgM-single positive and hIgM/CD21-double positive B cells seemed lower, except that percentages of hIgM/hIgκ (or hIgM/bIgκ)-double positive B cells likely increased in the cKSL-HACΔ series (i.e. isHAC, istHAC and cKSL-HACΔ) with hIgM/hIgλ-double positive B cells undetectable (data not shown). On the contrary, percentages of bIgM-single positive and bIgM/CD21-double positive B cells appear to be similar to those of the DKO background (Fig 9) in the KcHACΔ series (i.e. isKcHACΔ and KcHACΔ), with percentages of bIgM/hIgκ -double positive B cells remarkably increased.

**Table 1:** Production of cloned HAC/TKO calves from genetically modified fibroblast cell lines.

		Pregnant at (%)^a^				Calves(%)^a^
HAC	Recipients implanted	40 days	120 days	180 days	240 days	Live calves born
cKSL-HACΔ	549	175(32)	90 (16)	81(15)	71(13)	36(7)
KcHACΔ	655	258(40)	112 (17)	110(17)	100(15)	40(6)
isHAC	438	163(37)	67 (15)	57(13)	48(11)	20(5)
istHAC	435	179(41)	69 (16)	65(15)	61(14)	36(8)
isKcHACΔ	541	259(48)	108 (20)	103(19)	98(18)	42(8)
Total	2618	1034(40)	446 (17)	416(16)	378(14)	174(7)

^a^ Percentages were calculated by dividing the number of fetuses or calves by that of recipients implanted.

**Fig 11:**
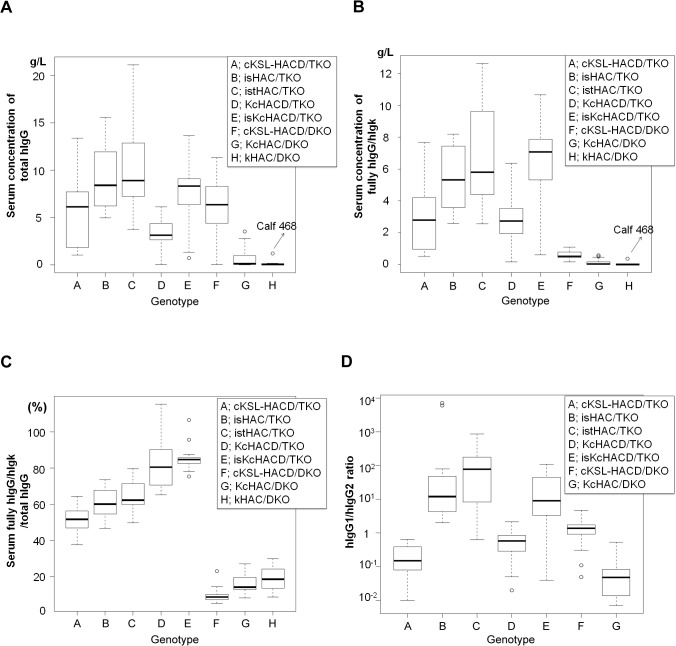
Characterization of isHAC/TKO, istHAC/TKO and isKcHACΔ/TKO cattle by serum IgG profile. (*A*) Box-whisker plots of serum concentrations of total hIgG (g/L) in a series of HAC/TKO and HAC/DKO cattle at 6 months of age. A, cKSL-HACΔ/TKO (n = 14); B, isHAC/TKO (n = 12); C, istHAC/TKO (n = 13); D, KcHACΔ/TKO (n = 20); E, isKcHACΔ/TKO (n = 17); F, cKSL-HACΔ/DKO (n = 43); G, KcHAC/DKO (n = 25); H, κHAC/DKO (n = 8). Dots represent outliers. The value of calf 468 was indicated with an arrow. (*B*) Box-whisker plots of serum concentrations of fully hIgG/hIgκ (g/L) in a series of HAC/TKO and HAC/DKO cattle at 6 months of age. A, cKSL-HACΔ/TKO (n = 13); B, isHAC/TKO (n = 12); C, istHAC/TKO (n = 13); D, KcHACΔ/TKO (n = 19); E, isKcHACΔ/TKO (n = 16); F, cKSL-HACΔ/DKO (n = 42); G, KcHAC/DKO (n = 21); H, κHAC/DKO (n = 8). Dots represent outliers. The value of calf 468 was indicated with an arrow. (*C*) Box-whisker plots of serum fully hIgG/hIgκ (%)/total hIgG in a series of HAC/TKO and HAC/DKO cattle at 6 months of age. A, cKSL-HACΔ/TKO (n = 13); B, isHAC/TKO (n = 12); C, istHAC/TKO (n = 13); D, KcHACΔ/TKO (n = 19); E, isKcHACΔ/TKO (n = 16); F, cKSL-HACΔ/DKO (n = 42); G, KcHAC/DKO (n = 21); H, κHAC/DKO (n = 3). Dots represent outliers. (*D*) Box-whisker plots of hIgG1/hIgG2 ratio in a series of HAC/TKO and HAC/DKO cattle at 6 months of age. A, cKSL-HACΔ/TKO (n = 14); B, isHAC/TKO (n = 12); C, istHAC/TKO (n = 13); D, KcHACΔ/TKO (n = 19); E, isKcHACΔ/TKO (n = 17); F, cKSL-HACΔ/DKO (n = 42); G, KcHAC/DKO (n = 12). Dots represent outliers. P values calculated for multiple comparison were shown in supplemental tables (S1, S2 and S3 Table).

Since our rationale for the isHAC, istHAC and isKcHACΔ vector construction was to directly determine how much the bovinization of the h*IGHG1* gene class switch regulatory element and transmembrane/cytoplasmic domains impacts on fully hIgG production profile under the bovine physiological condition, we measured serum concentrations of fully hIgG/hIgκ and hIgG subclass distribution at about 6 months of age in a series of HAC/TKO cattle (Fig 11A–11D). Overall, in all five HAC/TKO genotypes, both serum concentrations and percentages of fully hIgG/hIgκ substantially increased when compared with those of the HAC/DKO cattle, so the b*IGL* cluster deletion proved to be very effective for high production of fully hIgG/hIgκ in Tc cattle. Fully hIgG/hIgλ in the cKSL-HACΔ series (i.e. isHAC, istHAC and cKSL-HACΔ) was ~5% of hIgG/hIgκ (data not shown) and the rest was chimeric hIgG/bIgκ (S6 Fig). When compared with their original cKSL-HACΔ/TKO animals, fully hIgG/hIgκ production increased most significantly in istHAC/TKO cattle (*p* = 0.023, Fig 11B, S2 Table) while the isHAC/TKO did considerably, but not significantly. Of particular note, hIgG1 became a dominant subclass in both isHAC/TKO and istHAC/TKO cattle, whereas the original cKSL-HACΔ/TKO cattle are significantly turned hIgG2-dominant from hIgG1-dominancy in the cKSL-HACΔ/DKO ones (Fig 11D). Interestingly, this trend of observation was consistently seen also in comparison between the isKcHACΔ/TKO, KcHACΔ/TKO and KcHAC/DKO cattle, where fully hIgG/hIgκ production significantly increased (*p* <0.001 to the KcHACΔ/TKO and KcHAC/DKO cattle) (Fig 11B, S2 Table) with the significant switch to hIgG1-dominancy in the isKcHACΔ/TKO cattle from hIgG2-dominancy in the original KcHACΔ/TKO and KcHAC/DKO cattle (Fig 11A–11D).

These data demonstrated that the I_γ1_-S_γ1_ class switch regulatory element is controlled in a species-specific manner. The species-specific effect on fully hIgG serum concentration seems to be different between the differently bovinized cIgM proteins [cIgM(CH1) vs cIgM(CH2)]; the bovinization of the I_γ1_-S_γ1_ element in the cIgM(CH1) background significantly improved it (i.e. isKcHACΔ vs KcHACΔ) while it did not in the cIgM(CH2) background (i.e. isHAC vs cKSL-HACΔ). In the cIgM(CH2) background, the bovinization of IgG1 transmembrane/cytoplasmic domains was additionally necessary to significantly improve fully hIgG/hIgκ production (i.e. istHAC vs cKSL-HACΔ). Both in the cIgM(CH1) and cIgM(CH2) backgrounds, the bovinized I_γ1_-S_γ1_ sequence drastically altered hIgG1 subclass-dominancy.

**Fig 12:**
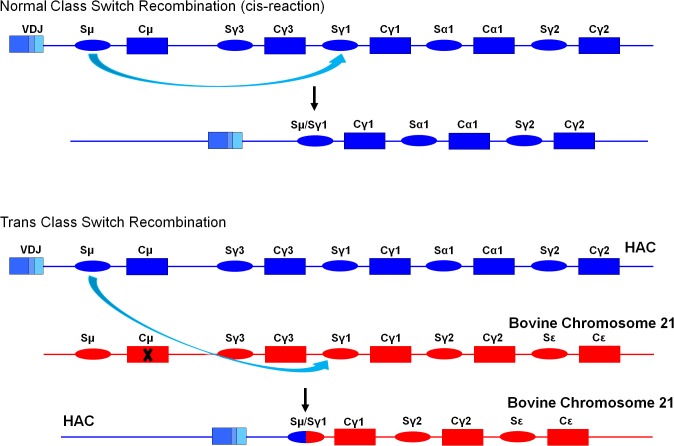
Generation of trans-class switched bovine IgG. Upper panel shows normal class switch recombination between human Sμ and human Sγ1 on the same chromosome (cis-reaction) in human or Tc bovine, which results in the expression unit of normal fully human γ1 heavy chain. The lower panel of the figure illustrates the trans-class switch recombination. Human Sμ on the HAC and bovine Sγ1 on the bovine chromosome 21 can make class switch recombination (trans-reaction), which results in the generation of expression unit with human VDJ connected to bovine gamma1 constant region.

**Fig 13:**
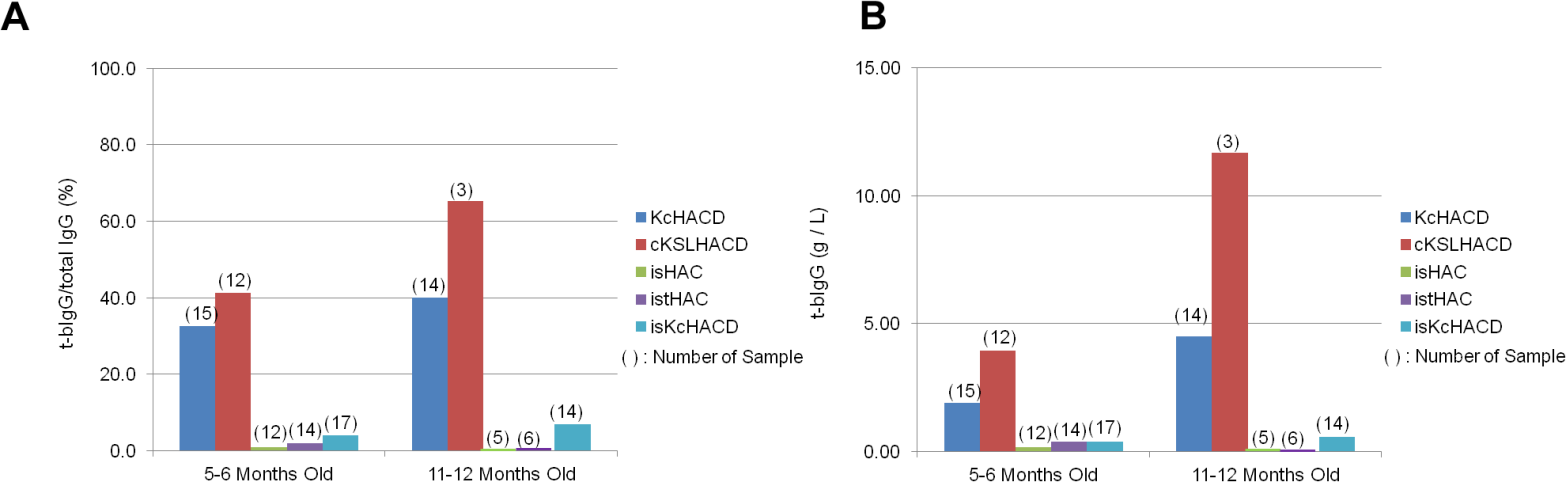
Trans-class switched bovine IgG levels in a series of Tc cattle. (A) The ratio of t-bIgG to total IgG (human IgG and t-bIgG) in percentage. The number of Tc **cattle** evaluated at each time points was noted on top of each bar in brackets. (B) Serum t-bIgG concentration in Tc bovines. The serum t-bIgG concentrations of Tc bovines with TKO background were evaluated at ages of 5–6 months and at 11–12 months. The number of Tc bovines evaluated at each time points was noted on top of the each bar in brackets.

Furthermore, bovinization of the I_γ1_-S_γ1_ class switch regulatory element resulted in drastic decrease in t-bIgG generation. T-bIgG can be generated by trans-chromosomal class switch recombination (Fig 12). This mechanism is supported by the sequence data of cloned genomic PCR products using forward primer located at 5’ of human Sμ region and the reverse primer located at 3’ of bovine Sγ1 region. The sequence data showed human Sμ connected to bovine Sγ1 (S7 Fig). Also cloned RT-PCR product using mixed human V gene sequence forward primers with bovine Cγ1 reverse primer showed functional transcript with human variable region with bovine Cγ1 sequence (S8 Fig). In order to evaluate the effect of bovinization of I_γ1_-S_γ1_ class switch regulatory element, we measured t-bIgG serum concentration in KcHACΔ, cKSL-HACΔ, isHAC, istHAC and isKcHACΔ with TKO background at 5–6 months old and 11–12 months old and calculated ratio of t-bIgG concentration to total IgG (total human IgG plus t-bIgG) concentration (Fig 13). Tc cattle without bovinization of I_γ1_-S_γ1_ class switch regulatory element (KcHACΔ, cKSL-HACΔ) showed high ratio of t-bIgG and high t-bIgG level in their serum, whereas Tc cattle with bovinization of I_γ1_-S_γ1_ class switch regulatory element (isHAC, istHAC and isKcHACΔ) showed clear reduction of t-bIgG (Fig 13A and 13B). These results indicate that species incompatibility in class switch regulatory element could be the cause of abnormal class switch recombination and bovinization of class switch regulatory element restored more efficient normal class switch recombination to human IgG1.

**Fig 14:**
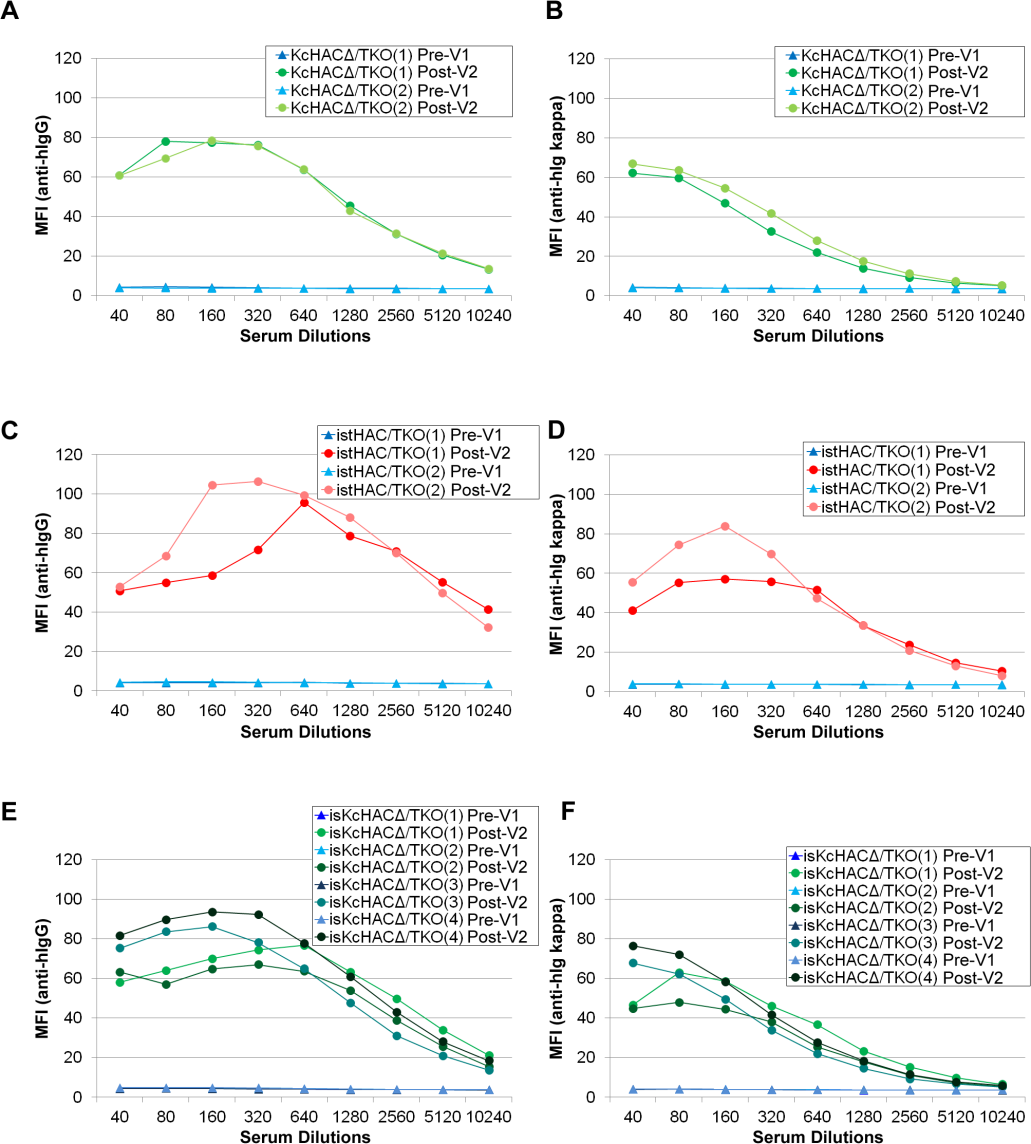
Anti-human carcinoma human IgG response in a series of Tc cattle. A series of HAC/TKO cattle received two times vaccinations (V2) of human oral squamous cell carcinoma and antigen-specific antibody generation was evaluated. Mean fluorescence intensity (MFI) of tumor cells stained with the sera from pre- and post-immunized cattle were plotted against a series of serum dilutions. Left panels (A, C, E) show the MFI with anti-hIgG antibodies (measuring total hIgG); right panels (B, D, F) show the MFI with anti-hIgκ antibodies (measuring fully hIgG/hIgκ). (A) and (B): KcHACD/TKO. (C) and (D): istHAC/TKO. (E) and (F): isKcHACD/TKO. Closed triangle and closed circle in the plot show MFI with sera from pre- and post- immunized cattle, respectively.

In order to elucidate if the HAC/TKO cattle that underwent such complex chromosome engineering (S9 Fig) could functionally generate fully hIgG/hIgκ pAbs in response to antigen immunization, we immunized several HAC/TKO cattle with human oral squamous cell carcinoma. All the HAC/TKO cattle immunized mounted robust anti-human carcinoma fully hIgG/hIgκ response (Fig 14). The data indicates that the HAC/TKO genotype is generally useful for high production of antigen-specific fully hIgG/hIgκ pAbs, which seemed to be further enhanced by the bovinized HAC vectors, istHAC and isKcHACΔ.

## Discussion

This study demonstrates a feasibility of producing high levels of fully hIgG/hIgκ (>5 g/L on average/median in novel genotypes, i.e. isHAC/TKO, istHAC/TKO and isKcHACΔ/TKO) in sera/plasma of a large farm animal species. This serum concentration of fully hIgG/hIgκ is, to our knowledge, the highest ever reported in in transgenic animals. For example, transgenic mice can only produce about 0.5g/L of serum fully hIgG [3, 11, 13]. The fully hIgG level is also the closest to or greater than that in healthy humans [14]. Moreover, hIgG subclass produced in isHAC/TKO, istHAC/TKO and isKcHACΔ/TKO cattle is hIgG1-dominant, which is the major subclass in healthy humans and is also widely used in therapeutic hIgG recombinant antibodies in development and on the market. Importantly, all the HAC/TKO cattle tested can functionally generate fully hIgG/hIgκ pAbs against human-origin antigens, which would be difficult to achieve by conventional human plasma-derived IVIG, due to immune tolerance in humans [15]. In order to reach this high production of fully hIgG/hIgκ pAbs in a large farm animal species, we exploited a unique approach, species-specific chromosome engineering, to address the interspecies-incompatibilities between human and bovine in IgG production machinery at multiple levels.

**Fig 15:**
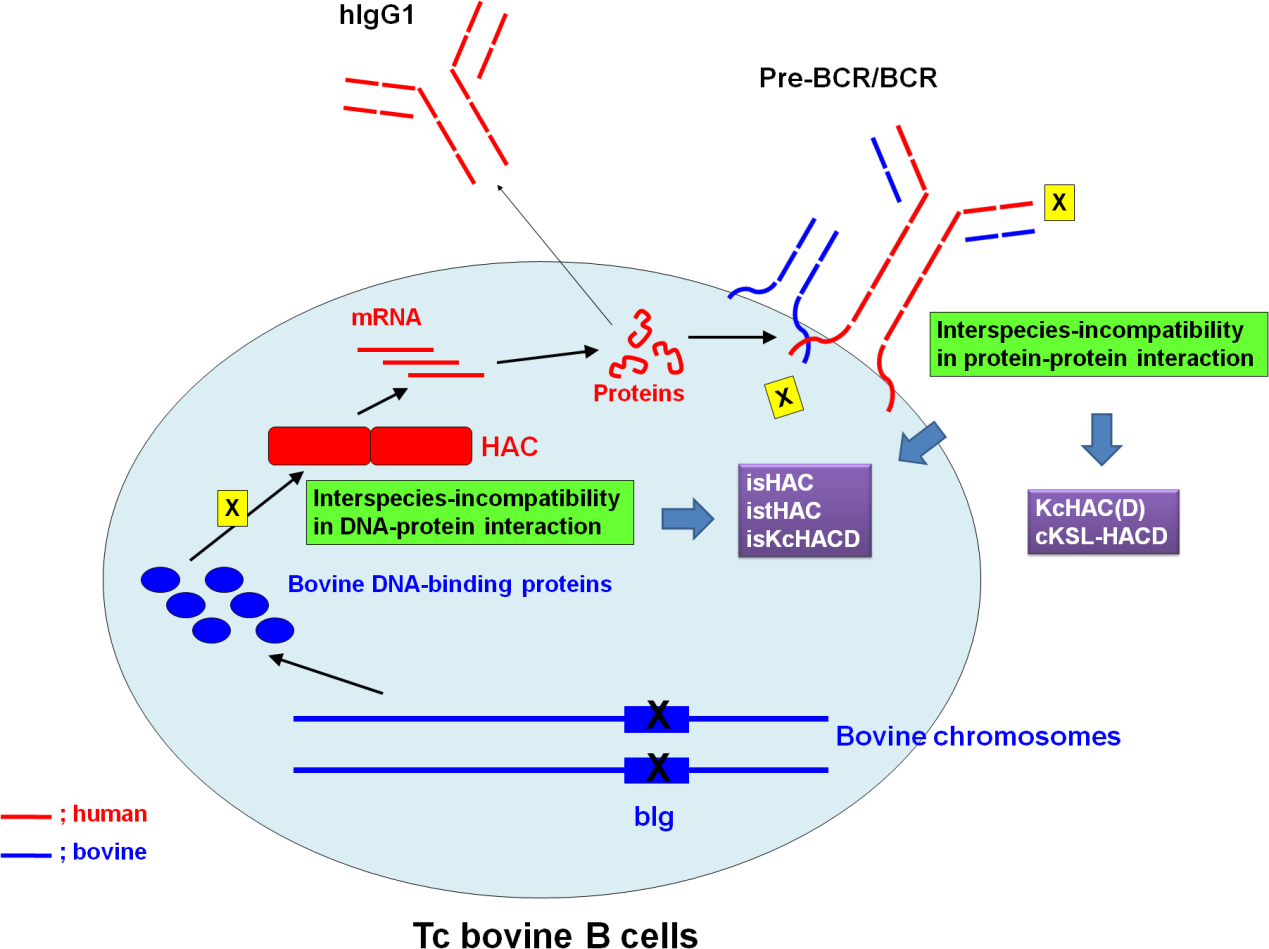
The proposed model of interspecies-incompatibilities at two levels in Tc bovine B cells. One is at protein-protein interaction, such as pre-BCR/BCR structure (e.g. pairing between human IgM and bovine surrogate/orthodox light chain, interaction between human IgM/IgG1 and bovine Ig-α/β). The other one is at DNA-protein interaction, such as between human I_γ1_-S_γ1_ DNA sequence and bovine cytokine/activator-induced bovine DNA binding proteins. The first interspecies-incompatibility is addressed by KcHAC(Δ) and cKSL-HACΔ (also by isHAC, istHAC and isKcHACΔ). The second interspecies-incompatibility is addressed by isHAC, istHAC and isKcHACΔ.

In our previous study [7], we investigated interspecies-incompatibilities at the level of protein-protein interaction (e.g. pre-BCR/BCR function); and in the current study, we explored this issue at the level of DNA-protein interaction (e.g. IgG1 class switch regulation) inTc bovine B cells (Fig 15). The interspecies-incompatibility at the level of protein-protein interaction was addressed by constructing the KcHACΔ (KcHAC) and cKSL-HACΔ vectors by bovinizing part of hIgM constant region without or with human SLC introduction, cIgM(CH1) or cIgM(CH2), respectively. Interestingly, the expression of these two distinct cIgM proteins on bovine B cells altered IgM B cell development considerably and the subsequent hIgG production profile significantly, as compared with the original κHAC vector [4]. While both vectors seemed to commonly increase the percentage of IgM/CD21-double positive B cells presumably by the better interaction with bovine Ig-α/β molecules in the DKO background, they behaved differently in the DKO vs TKO conditions; cIgM(CH1), such as KcHACΔ and isKcHACΔ, produces higher hIgM/hK than hIgM/bK in TKO background, whereas cIgM(CH2), such as cKSL-HACΔ, isHAC and istHAC, produces hIgM/hK and hIgM/bK at similar levels. These results correlated well with the ratio of fully human IgG to total human IgG in serum. The same trend about the lower fully human IgG ratio to the total human IgG in cIgM(CH2) Tc bovines than cIgM(CH1) Tc bovines can be observed in DKO background, too. Even though cIgM(CH2) with SLC increased total human IgG level significantly, Tc bovines with these modifications showed lower fully human IgG ratio to total human IgG than that from Tc bovines with cIgM(CH1) or wildtype IgM(based on the comparison to kHAC or KcHAC). On the other hand, cIgM(CH1) modification had little effect on fully human IgG ratio to total human IgG (based on the comparison between kHAC and KcHAC) and human SLC also had little impact on fully human IgG ratio to total human IgG (unpublished data).

In the DKO background, KcHAC [cIgM(CH1)] vector showed more obvious IgM/bIgλ, IgM/bIgκ and IgM/hIgκ-double positive B cells whereas either κHAC or cKSL-HACΔ [cIgM(CH2)] did not, indicating that the bovinized CH1 domain of the cIgM(CH1) from KcHAC vector might be critical to properly pair with bIgλ/bIgκ, presumably also with bovine SLC, especially through the bCλ/bCκ constant region and bovine λ5 of SLC. On the contrary, bCλ (or bCκ/bovine λ5) may not properly pair with the hCH1 domain due to the species-specific sequence difference. Another striking difference between the two cIgM proteins is hIgG1 subclass dominancy. The cIgM(CH1) revealed hIgG2-dominancy both in the DKO and TKO backgrounds whereas the cIgM(CH2) showed significantly distinct hIgG1-dominancy influenced by the presence of bIgλ expression (hIgG1-dominant in DKO vs hIgG2-dominant in TKO).

Taken together, these data suggest that such different features in IgM B cell development and hIgG production profile could be partly attributed to the distinct pre-BCR signaling; the cIgM(CH1) might more effectively pair with bovine SLC as it pairs better with bIgλ, whereas the cIgM(CH2) might preferentially pair with human surrogate light chain introduced by the hChr22 fragment as it did not properly pair with bIgλ (presumably not also with bovine surrogate light chain, either). This kind of different formats of IgM pre-BCR, pairing either with bovine or human SLC, might affect the following IgM BCR function, particularly BCR signal strength (this could be further influenced by pairing with bIgλ vs hIgκ), determining subsequent B cell fate, such as B1/marginal zone (MZ) B cell-like (T-independent responder) vs B2/follicular B cell-like (T-dependent responder), which is reported to eventually influence IgG subclass preference [16–21]; hIgG2 for T-independent vs hIgG1 for T-dependent response, respectively, in human [20, 21]. This is our first evidence on the interspecies-incompatibility in IgM pre-BCR/BCR function that eventually affects fully hIgG production profile.

The second evidence on the interspecies-incompatibility was revealed in IgG1 class switch regulation and production by bovinization of the h*IGHG1* gene class switch regulatory element, hI_γ1_-hS_γ1_, with or without the h*IGHG1* gene transmembrane/cytoplasmic domains also being bovinized. Notably, the isHAC/TKO, istHAC/TKO and isKcHACΔ/TKO cattle significantly altered hIgG1 production profile in comparison with their original cKSL-HACΔ/TKO and KcHACΔ/TKO cattle which were both hIgG2-dominant. Some considerable differences observed between the isHAC/TKO and istHAC/TKO cattle may suggest another interspecies-incompatibility in IgG1 transmembrane/cytoplasmic domains function such that the bovinized IgG1 transmembrane/cytoplasmic domains could be more compatible with bovine signal transduction molecules for the higher productivity of fully hIgG/hIgκ with hIgG1 subclass being dominant in the bovine condition. This is consistent with a report that IgG1 transmembrane/cytoplasmic domains function is critical for IgG1 immunoglobulin secretion [22].

The effect of the bovinized I_γ1_-S_γ1_ sequence is of a particular interest. It is reported that virtually all transcription factor-binding locations, landmarks of transcription initiation, and the resulting gene expression observed from the hChr21 in the human hepatocytes were recapitulated across the entire hChr21 in the mouse hepatocyte nucleus [23]. This implies that the human-specific gene expression profile could be simply provided by the human DNA primary sequence even under the non-human species environment. If we apply this view to our Tc bovine situation, the non-bovinized HAC (i.e., human DNA primary sequence) would have been mostly sufficient for providing the human-like hIgG expression profile, such as hIgG1-dominancy, in the bovine condition. However, this was not the case. Thus, our finding that bovinization of the hI_γ1_-hS_γ1_ sequence sufficiently caused the significant switch from high t-bIgG to very low t-bIgG and also from hIgG2-dominancy to hIgG1-dominancy in the Tc bovine condition strongly suggests the interspecies-incompatibility in IgG1 class switch regulation between the two species.

In summary, “functional chromosomics” using HAC vectors identified species-specific genomic elements (i.e. pre-BCR/BCR components and IgG1 class switch regulatory element, I_γ1_-S_γ1_) that greatly improve fully hIgG production profile at multiple levels in Tc cattle. These interspecies-sequence differences are responsible for low production of fully hIgG in our early versions of Tc cattle. The species-specific modifications thereof resulted in much higher production of fully hIgG, which is practically an important milestone towards human therapeutic applications beyond current human plasma-derived IVIG therapies. What is also important is to note that this complex chromosome engineering was done in somatic fibroblast cells, which are available in virtually any species, to alleviate a necessity of using ES cells. Therefore, this approach may be broadly applicable for more complicated and dynamic functional genomics at a chromosome level [24] in non-murine mammalian species for basic science and biomedical/agricultural applications.

This project was initiated more than 10 years ago with the concept of developing a platform technology to produce a wide range of human antibody products at scale using large farm animals such as cattle. The current report clearly proved that there is a great potential to use Tc bovine system for the clinical applications in the future.

## Methods

### Ethics statement

The animal protocols contained in the study were approved by the Hematech (previous name of SAB Biotherapeutics, Inc.) Institutional Animal Care and Use Committee (IACUC) (USDA Research Facility 46-R-0008, OLAW #A4438-01, AAALAC #001114). Care of all vertebrate animals was subject to regular review by the IACUC and complied with Animal Welfare laws and regulations of the United States. Periodic health evaluations (blood profile, weight, etc.) were made by Veterinary Service to ensure that Tc cattle were healthy. All Tc bovine received adequate housing, feed, access to water and bedding. Daily observations were made by herdsmen to ensure that appropriate standards of animal care were being met. SAB Biotherapeutics, Inc. uses *the Guide for the Care and Use of Laboratory Animals* and *the Guide for the Care and Use of Agricultural Animals Used in Agricultural and Research Teaching* for animal care standards. Animal care and use and facilities were inspected by the IACUC on a semiannual basis. Animals may have to be euthanized due to unfortunate events like terminal illness and trauma. Methods of euthanasia followed the guidelines given by the American Veterinary Medical Association (AVMA) 2013. These are acceptable methods by the AVMA and they were approved by the IACUC. All protocols and procedures used in the Animal Care and Use Program that may have caused discomfort, distress and pain, and injury were reviewed by the IACUC for appropriate management practices. Analgesic, anesthetics and tranquillizing drugs were used when appropriate under supervision of Veterinary Services. Veterinarians and herdsmen used cattle chute for restraining when needed. Cattle were not restrained for more than four hours per day.

### Genomic library

Genomic library from CHO cells containing the κHAC vector (κC1-1) [7] or the bovine fibroblast cell lines [4] were prepared as described previously. Library screening and λ phage DNA extraction/purification was performed as described previously [4]. The bovine genomic BAC library (CHORI-240) was purchased from Children’s Hospital Oakland Research Institute and screening was performed according to their instruction.

### Construction of targeting vectors

Each vector construction was performed as previously described [4, 25–27] with some modifications.

pCH1CAGzeo(R)DT(F): a genomic λ phage library was constructed from CHO cells containing the κHAC using λFIX II vector through a custom library construction service (Lofstrand). The genomic library was screened for h*IGHM* constant region by using a probe that was a PCR product by amplified a PCR pair, hCμ-FR, and then clones #1, #4 and #7 were isolated. From clone #4, 1.7 kb of *Pml* I fragment was subcloned into *Sma* I site of pBluescript, generating pCH1S (F). 1 kb of *Sac* I-*Pml* I fragment from plasmid pBCμAY37-95 where *Sal* I-bovine *IGHM* genomic fragment was cloned into pBluescript was subcloned into *Pst* I site of the pCH1S (F), generating pCH1SSP (F). 7.4 kb of the *Sma* I-*Eco* RI fragment from the above clone #1 was cloned into *Eco* RV/*Eco* RI-digested pCH1SSP (F), generating pCH1SL. On the other hand, from the plasmid pBCμAY37-95, 3.5 kb of *Sac* I fragment was subcloned into pBluescript and then the *Xho* I fragment of floxed CAGzeo [CAGzeo fragment was subcloned into *Eco* RV site of pBS246 (Gibco)] was cloned into *Van91* I site, generating pmAYSazeo (F). The *Sac* I fragment from the pmAYSazeo (F) was further subcloned into blunted *Eco* RI site of pCH1SL, generating pCH1zeo (F). As a final step, the *DT-A* cassette was subcloned into *Not* I site of the pCH1zeo (F) to complete the pCH1CAGzeo(R)DT(F).

pCC1BAC-isHAC: the genomic λ phage library constructed from CHO cells containing the κHAC was screened to isolate genomic DNA fragments covering the human I_γ1_-S_γ1_ region followed by the h*IGHG1* constant region by using a probe that was a PCR product by amplified with a PCR pair, g1(g2)-FR, and then we identified clones #h10 and #h18/h20. From clone #h10, 2 kb of *Afe* I-*Bam* HI fragment was rescued to be used as a short arm while 10.5 kb of *Apa* I-*Hpa* I fragment was obtained from clone #h18/h20 for a long arm. On the other hand, a bovine genomic λ phage library was screened to isolate genomic DNA fragments covering the bovine I_γ1_-S_γ1_ region followed by the b*IGHG1* constant region by using a probe that was a PCR product by amplified with a PCR pair, bIgG1-FR, and then we identified a clone #b42, from which a 9.7 kb fragment (5’ end through *Bsu36* I) was assembled to replace a 6.8 kb of the human I_γ1_-S_γ1_ region. A *Bsu*36 I-*Apa* I linker was used to join 3’ end of the bovine I_γ1_-S_γ1_ region and 5’ end of the h*IGHG1* constant region. The *neo* gene flanked by *FRT* and *DT-A* gene were inserted as shown in S5 Fig All the above assembles were done on a BAC-based backbone vector pCC1BAC (EPICENTRE) (S5 Fig).

phI_γ1_FRTCAGattPhisDDT: 11.4 kb of *Kpn* I-*Not* I genomic fragment from clone h10 was isolated from the clone #h10 and subcloned into pBluescript SK(–) vector. Then, the *FRT*-CAG promoter-*attP*-polyA-*hisD* cassette was inserted into the 5’ *Bam* HI site which is 1.8 kb downstream from the *Kpn* I site. Finally, *DT-A* gene was cloned into *Not* I site.

ph_γ1_TMNeoattPDT: 7.5 kb of *Sac* II genomic fragment from clone h20 was subcloned into pBluescript SK (–) vector. Next, the *neo*-*attP* cassette was inserted into *Hin* dIII site, followed by cloning of *DT-A* gene into *Not* I site.

pBAC-istHAC: 7.3 kb of *Bmg* BI-*Sph* I bovine genomic fragment containing the bovine TM1/TM2 domain was obtained from the clone #b66, of which 5’ part was joined with 3’ part of the 9.5 kb of the bovine I_γ1_-S_γ1_ fragment (from #b42) and 1.6 kb of h*IGHG1* gene (from #h10) from the isHAC by a linker, pNsiI-bG1-hG1-BmgBI. The *attB*-DsRed-*FRT* cassette was inserted at 5’ side of the 9.5 kb of the bovine I_γ1_-S_γ1_ fragment (from #b42) and another *attB* sequence was placed at 3’ side of 7.3 kb of *Bmg* BI-*Sph* I bovine genomic fragment containing the bovine TM1/TM2 domain that was obtained from the clone #b66. All the above assembles were done on a BAC-based backbone vector pCC1BAC (EPICENTRE) (S5 Fig).

### Transfection of chicken DT40 cells for HAC vector construction

HAC vector construction was carried out as previously described [4, 7].

### Genomic PCR and RT-PCR analyses

These analyses were implemented as previously described [4, 26]. All the PCR products were run on 0.8% agarose gels. Primer sequences are available from Supplementary information (S10 Fig).

### CGH analysis

Array probes for CGH analysis were designed by Roche NimbleGen, based on estimated sequence of the cKSL-HACΔ vector (see S11 Fig). Experiments and data analysis were performed by Roche NimbleGen.

### FISH analysis

Human COT-1 FISH and hChr-specific multi-color FISH were performed as previously described [4]. To specifically stain the h*IGH*, h*IGK* and h*IGL* loci, probes were synthesized from DNA derived from BAC clones RP11-417P24, RP11-316G9 and RP11-22M5, respectively.

### Flow cytometry analysis

Flow cytometry analysis on B cell development in newborn Tc calves were performed as previously described [4].

### ELISA

Total hIgG, fully hIgG/hIgκ, hIgG/hlgλ, hIgG/bIgκ, hIgG1 and hIgG2 ELISA assays were performed as previously described [4].

### Radial immunodiffusion assay

**T**-Bovine IgG levels in Tc bovine sera were measured by using the radial immunodiffusion kit from Triple J Farms in accordance with manufacturer’s instructions.

### Immunization of Tc cattle with human oral squamous cell carcinoma antigen and titer assay

HAC/TKO and HAC/DKO cattle were immunized with X-ray-irradiated human oral squamous cell carcinoma (DSMZ) antigen and serum samples were collected as previously described [4, 7] after each immunization for antibody titer analysis. Anti-human oral squamous cell carcinoma antibody titers were determined by flow cytometry analysis as described previously [7].

### Somatic cell nuclear transfer

Cloned fetuses and calves were produced using chromatin transfer procedure as described previously [4, 25–27].

### Statistical analysis

R version 3.0.2 was used for all statistical analysis under Mac OS X 10.6.8 environment. Due to the nature of data, unbalanced (unequal number of observations per treatment) and unequal variances (i.e., heteroscedastic) as can be seen from the box-whisker plots, an add-on R-package for performing multiple comparisons called multcomp [28] (version 1.3–2) in conjunction with the sandwich estimator for covariances [29, 30] (sandwich, version 2.3–0) were used to generate the adjusted *p*-values and the graph of the simultaneous confidence intervals for all the pairs.

## Supporting Information

S1 Fig Screening and confirmation of CH1D clones [cIgM(CH1) modified 14D basal vector]. (A) PCR screening for CH1D clones. (B) FISH analysis for selected clones #2, 3, 9, 10.
(TIF)

S2 Fig Screening and confirmation of KCD clones (CH1D-Z7 hybrid cells line). (A) PCR screening for clones with both human chromosome 14 fragment (CH1D) and chromosome 2 fragment (Z7). (B) FISH analysis for selected clones #1 and #34.
(TIF)

S3 Fig Analysis of FACS sorted GFP positive samples for KcHACΔ construction. (A) PCR analysis of FACS sorted GFP low expresser. (B) FISH analysis of FACS sorted GFP high and low populations.
(TIF)

S4 Fig Alignment of the sequences relevant to IgG1 class switch regulation and secretion between bovine and human. (A) Alignment of DNA sequences of the Iγ1 (Igamma 1), Iγ2 (Igamma 2) and Iγ3 (Igamma 3) ECS (evolutionary conserved sequence) elements between human and bovine. Nucleotide bases in red boxes depict differences from the human Iγ1 sequence. Binding sites of KB3, KB4, KB5, ISRE, C-EBP, BSAP and GAS are indicated by red-line rectangle. (B) Amino acid sequence alignment of the IgG1 transmembrane/cytoplasmic domains between human and bovine. Amino acids in red boxes depict differences from human.
(TIF)

S5 Fig Detailed information of targeting vectors pCC1BAC-isHAC and pBAC-istHAC. The bovinizing vector pCC1BAC-isHAC is a BAC-based one (backbone is pCC1BAC vector), consisting of 10.5 kb and 2 kb of genomic DNA as a long and short arm, respectively, 9.7 kb of the bovine genomic DNA covering the bovine Iγ1-Sγ1 and its surrounding region to replace the human corresponding 6.8 kb of Iγ1-Sγ1 region, the chicken β-actin promoter-driven *neo* gene flanked by *FRT* sequence and the *DT-A* gene. The 2 kb of Afe I-Bam HI fragment and 10.5 kb of Apa I-Hpa I fragment for a short arm and long arm were obtained from clone h10 and clone h18/h20, respectively, derived from λ phage genomic library constructed from CHO cells containing the κHAC by screening with a probe around the human Iγ1-Sγ1 region. The 9.7 kb fragment (5’ end through Bsu36 I) was obtained from clone b42 derived from the λ phage bovine genomic library. Another bovinizing vector pBAC-istHAC is also the same BAC-based vector, which consists of the above 9.7kb bovine genomic fragment and human C1 1.6kb genomic fragment and 7.3kb bovine TM region containing genomic fragment, and these fragments were connected by Bsau36I-ApaI linker and pNsiI-bG1-hG1-BmgBI linker on BAC. These genomic fragments were flanked with *attB*-DsRed-*FRT* cassette and *attB* cassette at 5’end and 3’end respectively. These three genomic fragments were obtained from phage clones of the above explained two genomic libraries (b42, h10 and b66).
(TIF)

S6 Fig Serum hIgG/bIgκ (%)/total hIgG in a series of HAC/TKO and HAC/DKO cattle at 5–6 months of age. n, number of Tc bovines analyzed for each genotype. For each genotype, values of minimum, first quartile, median, third quartile and maximum were calculated and plotted in the graph.
(TIF)

S7 Fig Example of human Sμ-bovine Sγ1 junction sequence. Genomic PCR product with primers tCSR-F1R1 and genomic DNA derived from blood of a Tc bovine (KcHAC/DKO) was cloned into pGEM-T vector (Promega) for sequencing. The sequence in this figure is from a plasmid clone. This is an example of human Sμ and bovine Sγ1 junction sequence which is derived from trans-class switch recombination between human Sμ and bovine Sγ1.
(TIF)

S8 Fig Example of t-bIgG transcripts. RT-PCR product with forward mixed primers for human VH gene and reverse primer which is specific to bovine IgG constant region was cloned into pGEM-T vector (Promega) and sequenced. This figure shows two examples of cloned RT-PCR products derived from two Tc bovines (one kHAC/DKO and one KcHAC/DKO). Both of them showed functional VDJ sequence connected to bovine Cγ1 in frame.
(TIF)

S9 Fig Detailed structure of isKcHACΔ, isHAC and istHAC. Chr.2, Chr.14 and Chr.22 with the following numbers show the location in the public database sequences from NC_000002.11, for human chromosome 2, NC000014.8 for human chromosome 14 and NC_000022.10 for human chromosome 22, respectively.
(TIF)

S10 Fig List of oligo DNA sequences. Oligo DNA sequences used for linker or PCR primer in this study were listed with their names and sequences.
(XLS)

S11 Fig Probe design for CGH, based on the deduced cKSL-HACΔ vector sequence. For hChr14, hChr2 and hChr22 fragment sequences, the AB019437 to AL512355, the AC113612 to AC104134, and the AP000553 to AP000350, respectively, were assembled and linked with artificial “NNN…N” to create the 4,932,030 bp DNA sequence as the deduced cKSL-HACΔ vector sequence.
(TIF)

S1 Table *p* values for the comparison of serum total hIgG concentrations among different genotypes (DOCX)

S2 Table *p* values for the comparison of serum fully hIgG/hIgκ concentrations among different genotypes (DOCX)

S3 Table *p* values for the comparison of serum fully hIgG/hIgκ (%)/total hIgG among different genotypes (DOCX)
