# Supplementary figures and images for: Species-Specific Chromosome Engineering Greatly Improves Fully Human Polyclonal Antibody Production Profile in Cattle
Source: PLoS One. 2015 Jun 24;10(6):e0130699. doi: 10.1371/journal.pone.0130699 (PMC4479556; doi:10.1371/journal.pone.0130699)

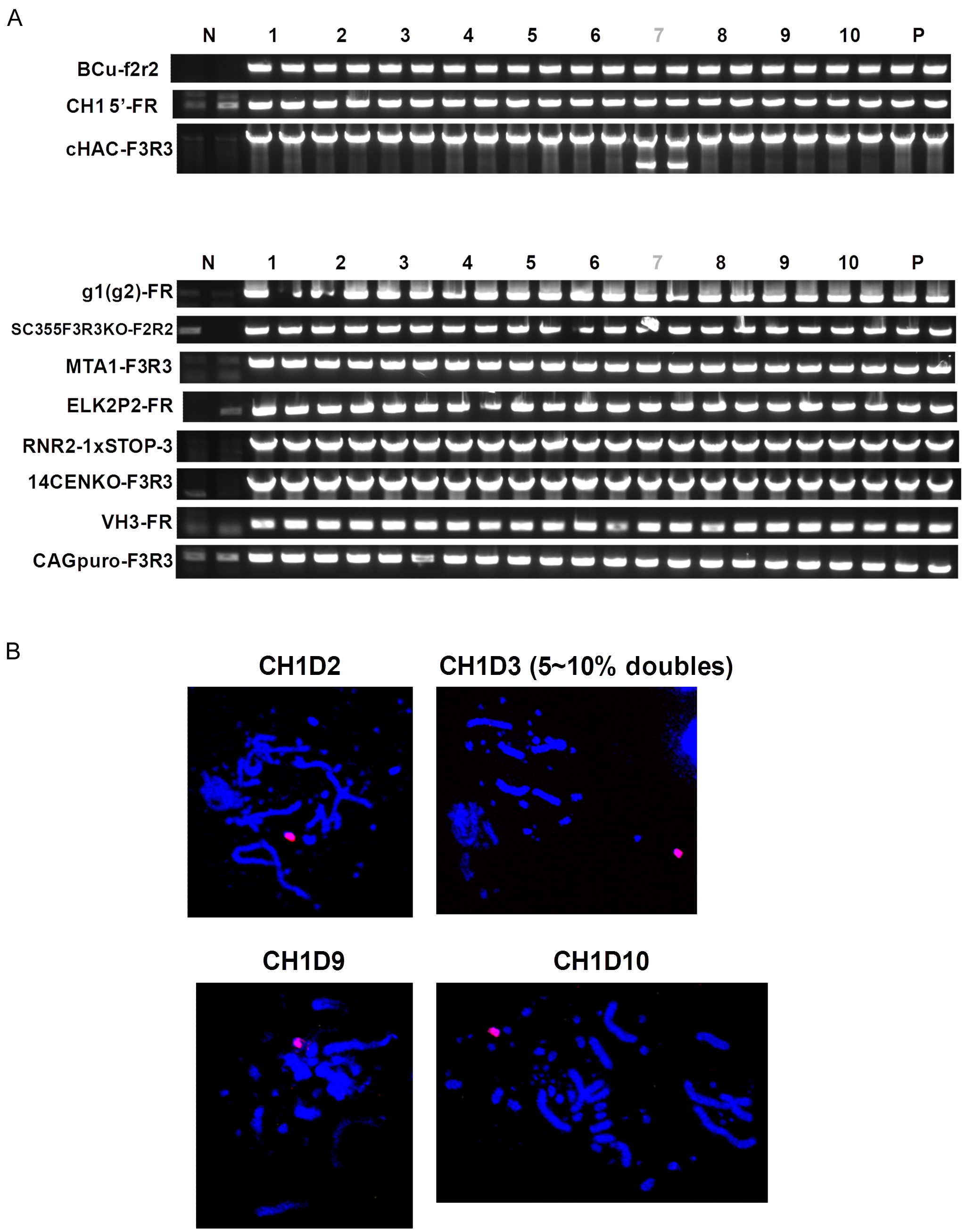

Supplement: S1 Fig — (A) PCR screening for CH1D clones. (B) FISH analysis for selected clones #2, 3, 9, 10. (TIF) [file pone.0130699.s001.tif]

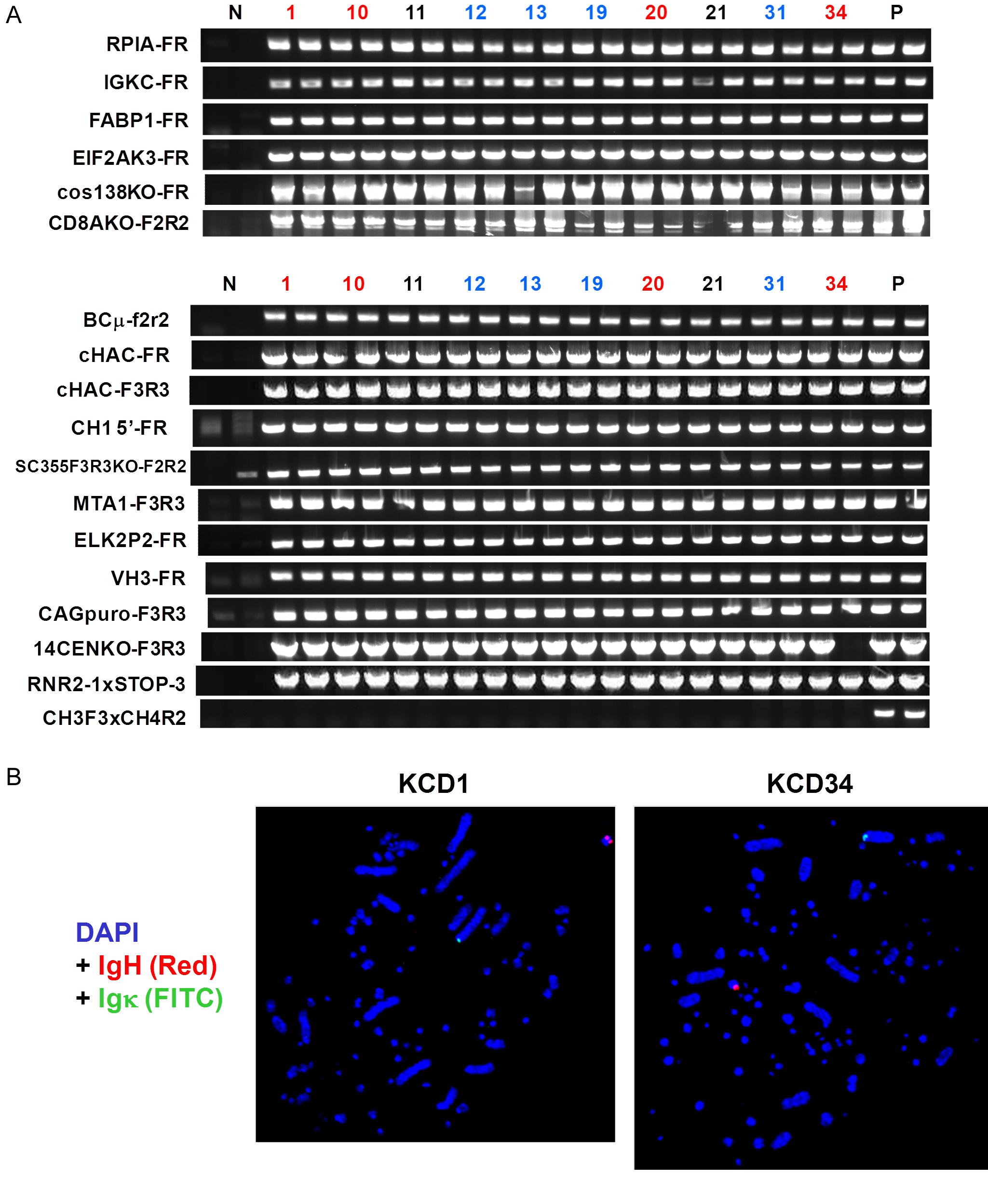

Supplement: S2 Fig — (A) PCR screening for clones with both human chromosome 14 fragment (CH1D) and chromosome 2 fragment (Z7). (B) FISH analysis for selected clones #1 and #34. (TIF) [file pone.0130699.s002.tif]

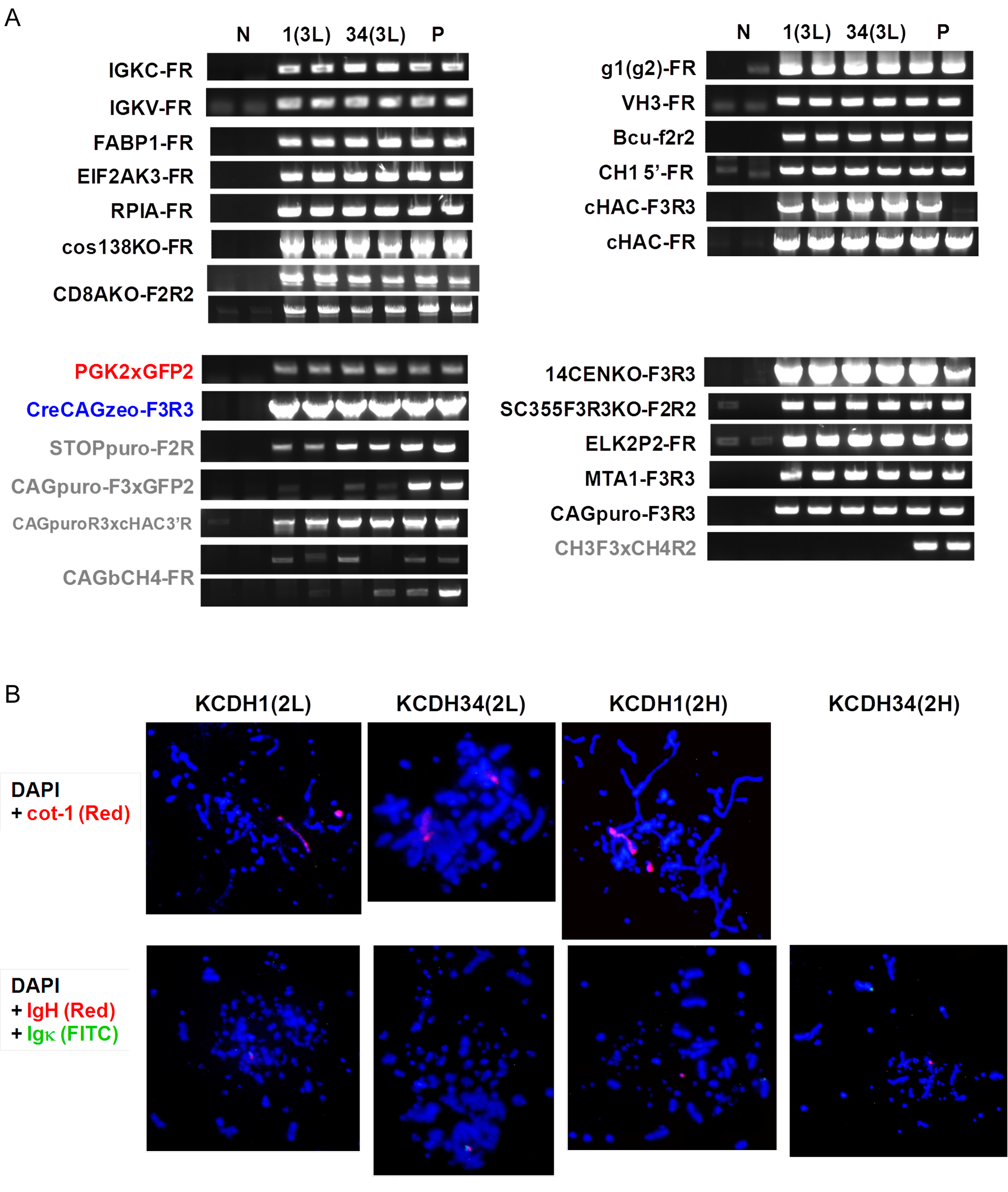

Supplement: S3 Fig — (A) PCR analysis of FACS sorted GFP low expresser. (B) FISH analysis of FACS sorted GFP high and low populations. (TIF) [file pone.0130699.s003.tif]

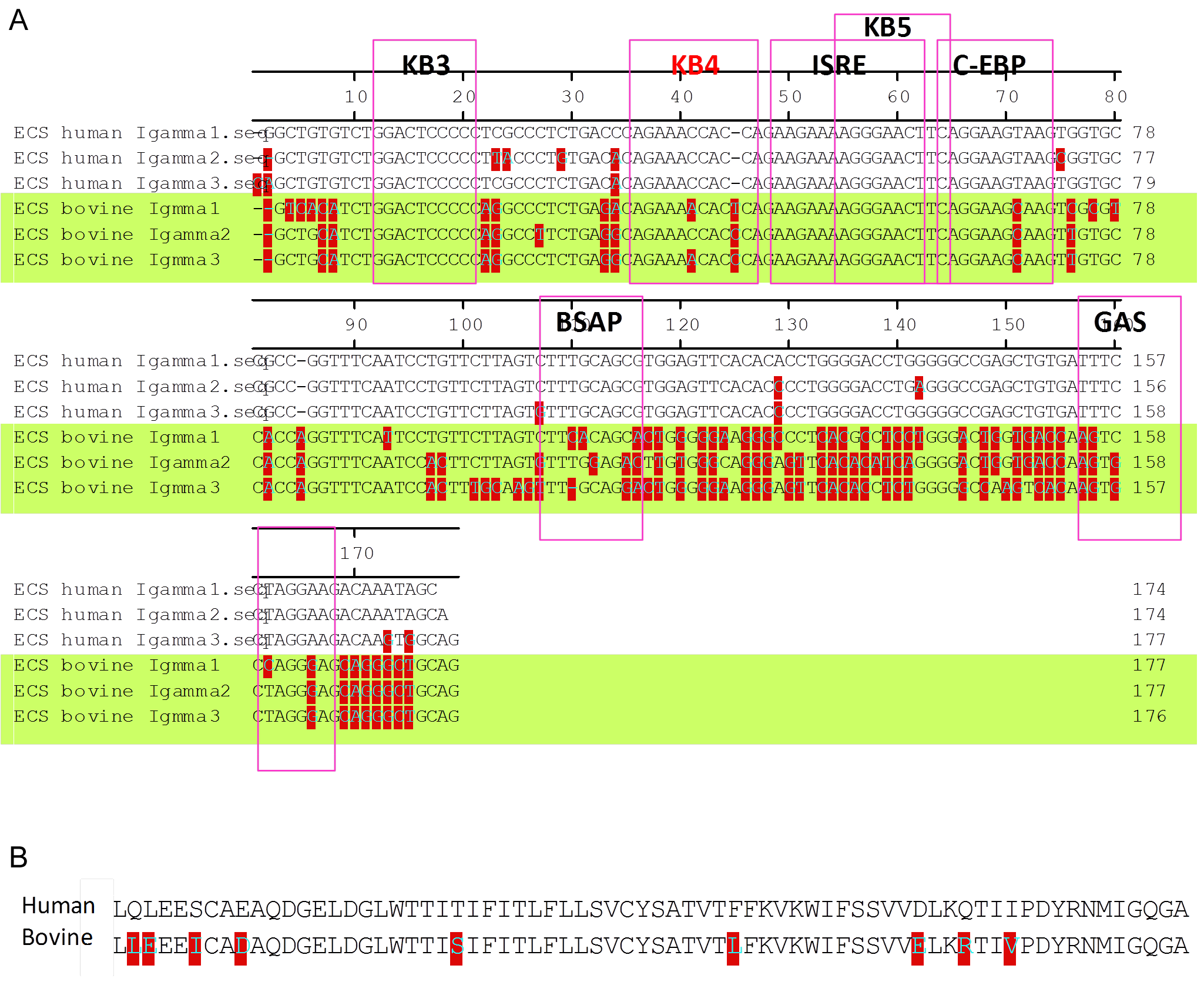

Supplement: S4 Fig — (A) Alignment of DNA sequences of the Iγ1 (Igamma 1), Iγ2 (Igamma 2) and Iγ3 (Igamma 3) ECS (evolutionary conserved sequence) elements between human and bovine. Nucleotide bases in red boxes depict differences from the human Iγ1 sequence. Binding sites of KB3, KB4, KB5, ISRE, C-EBP, BSAP and GAS are indicated by red-line rectangle. (B) Amino acid sequence alignment of the IgG1 transmembrane/cytoplasmic domains between human and bovine. Amino acids in red boxes depict differences from human. (TIF) [file pone.0130699.s004.tif]

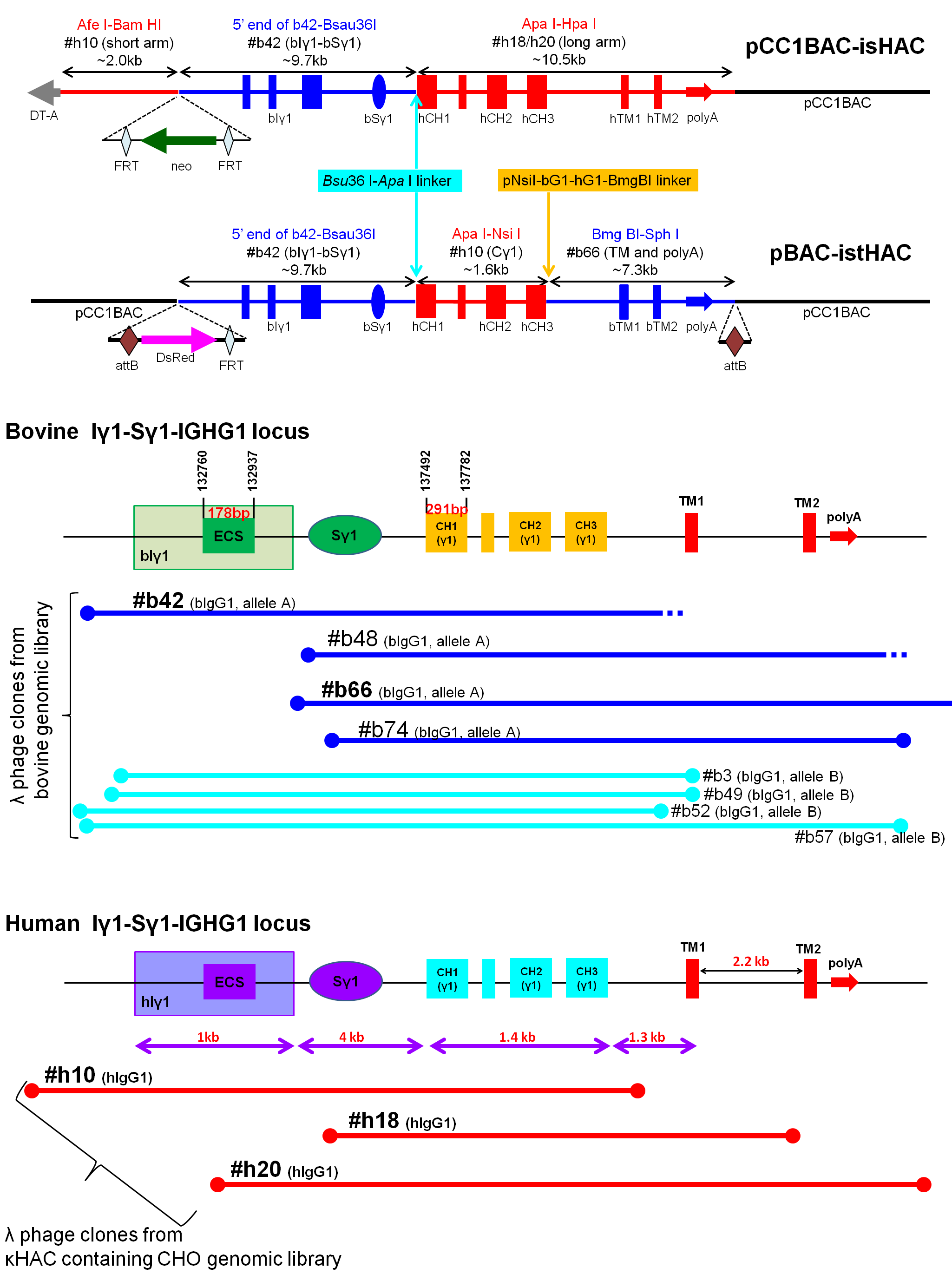

Supplement: S5 Fig — The bovinizing vector pCC1BAC-isHAC is a BAC-based one (backbone is pCC1BAC vector), consisting of 10.5 kb and 2 kb of genomic DNA as a long and short arm, respectively, 9.7 kb of the bovine genomic DNA covering the bovine Iγ1-Sγ1 and its surrounding region to replace the human corresponding 6.8 kb of Iγ1-Sγ1 region, the chicken β-actin promoter-driven neo gene flanked by FRT sequence and the DT-A gene. The 2 kb of Afe I-Bam HI fragment and 10.5 kb of Apa I-Hpa I fragment for a short arm and long arm were obtained from clone h10 and clone h18/h20, respectively, derived from λ phage genomic library constructed from CHO cells containing the κHAC by screening with a probe around the human Iγ1-Sγ1 region. The 9.7 kb fragment (5’ end through Bsu36 I) was obtained from clone b42 derived from the λ phage bovine genomic library. Another bovinizing vector pBAC-istHAC is also the same BAC-based vector, which consists of the above 9.7kb bovine genomic fragment and human C1 1.6kb genomic fragment and 7.3kb bovine TM region containing genomic fragment, and these fragments were connected by Bsau36I-ApaI linker and pNsiI-bG1-hG1-BmgBI linker on BAC. These genomic fragments were flanked with attB-DsRed-FRT cassette and attB cassette at 5’end and 3’end respectively. These three genomic fragments were obtained from phage clones of the above explained two genomic libraries (b42, h10 and b66). (TIF) [file pone.0130699.s005.tif]

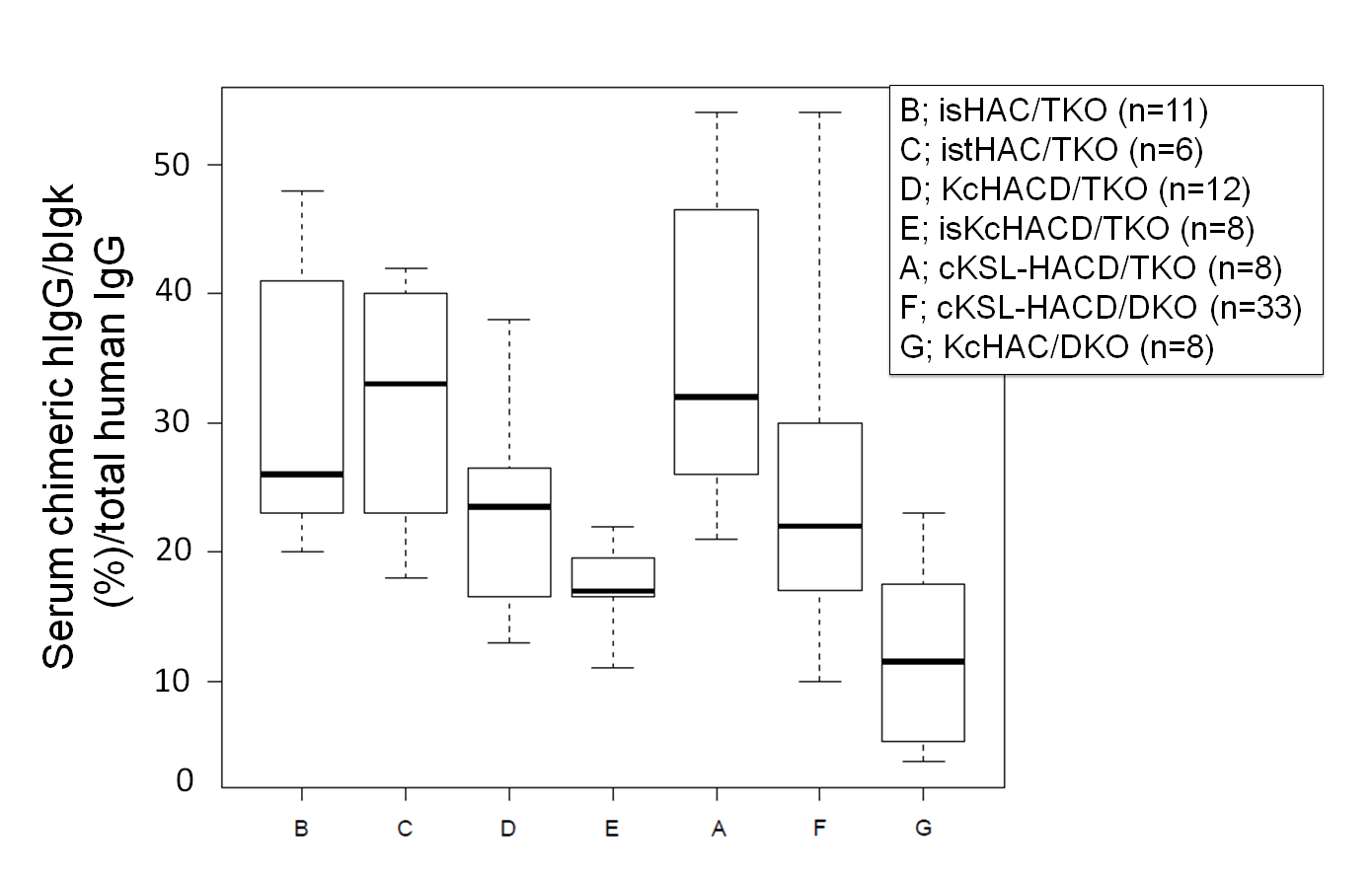

Supplement: S6 Fig — n, number of Tc bovines analyzed for each genotype. For each genotype, values of minimum, first quartile, median, third quartile and maximum were calculated and plotted in the graph. (TIF) [file pone.0130699.s006.tif]

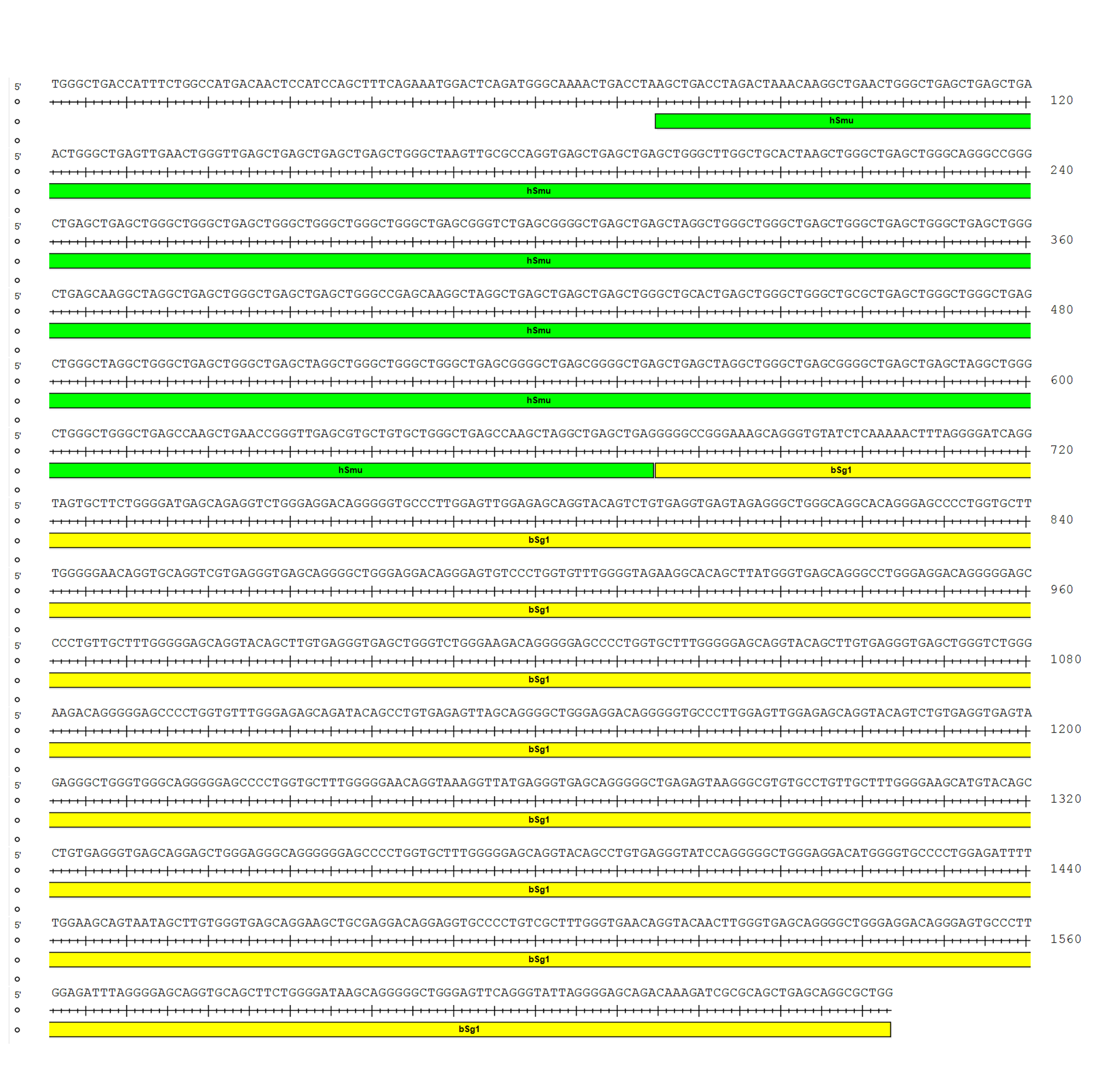

Supplement: S7 Fig — Genomic PCR product with primers tCSR-F1R1 and genomic DNA derived from blood of a Tc bovine (KcHAC/DKO) was cloned into pGEM-T vector (Promega) for sequencing. The sequence in this figure is from a plasmid clone. This is an example of human Sμ and bovine Sγ1 junction sequence which is derived from trans-class switch recombination between human Sμ and bovine Sγ1. (TIF) [file pone.0130699.s007.tif]

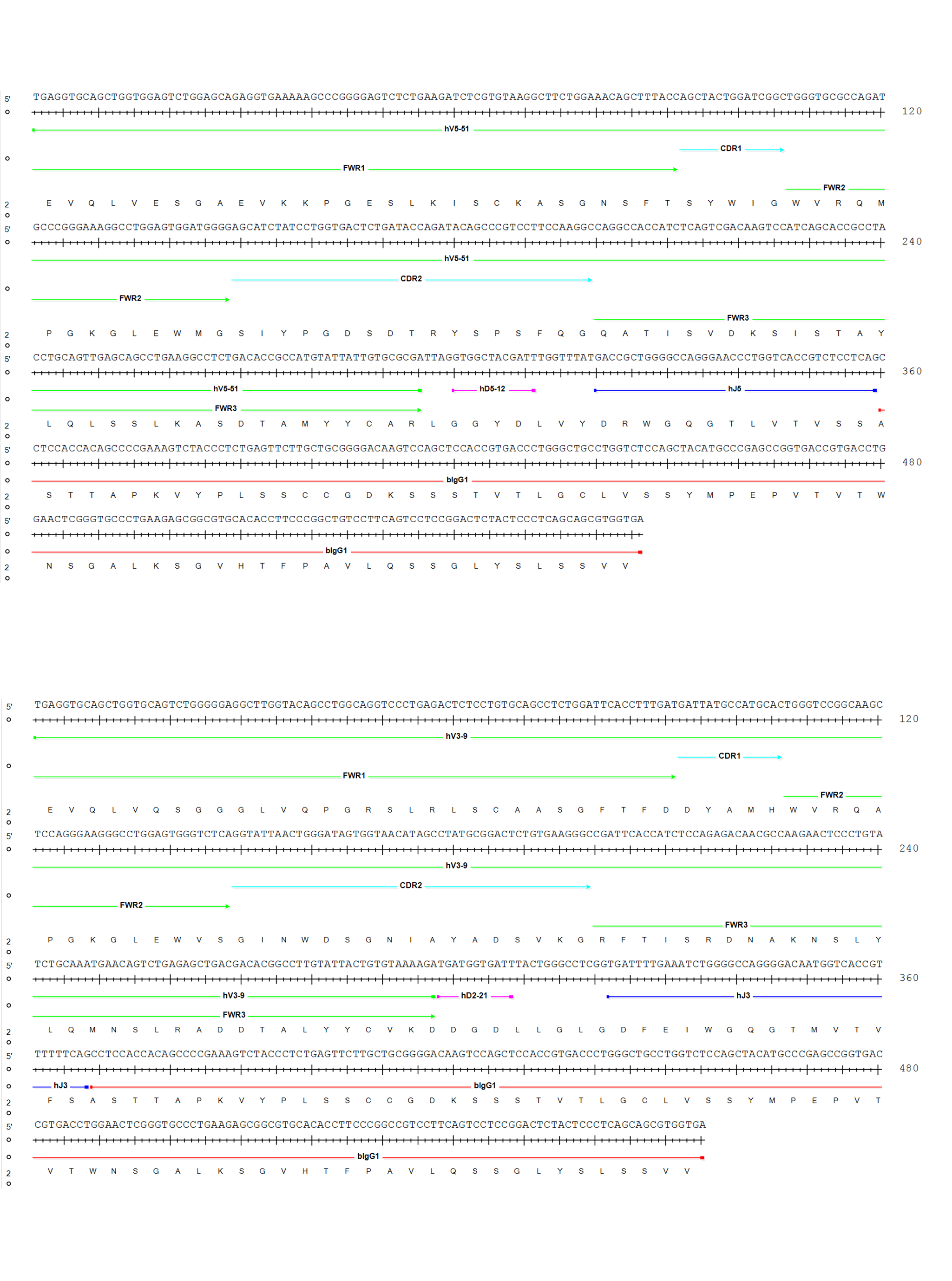

Supplement: S8 Fig — RT-PCR product with forward mixed primers for human VH gene and reverse primer which is specific to bovine IgG constant region was cloned into pGEM-T vector (Promega) and sequenced. This figure shows two examples of cloned RT-PCR products derived from two Tc bovines (one kHAC/DKO and one KcHAC/DKO). Both of them showed functional VDJ sequence connected to bovine Cγ1 in frame. (TIF) [file pone.0130699.s008.tif]

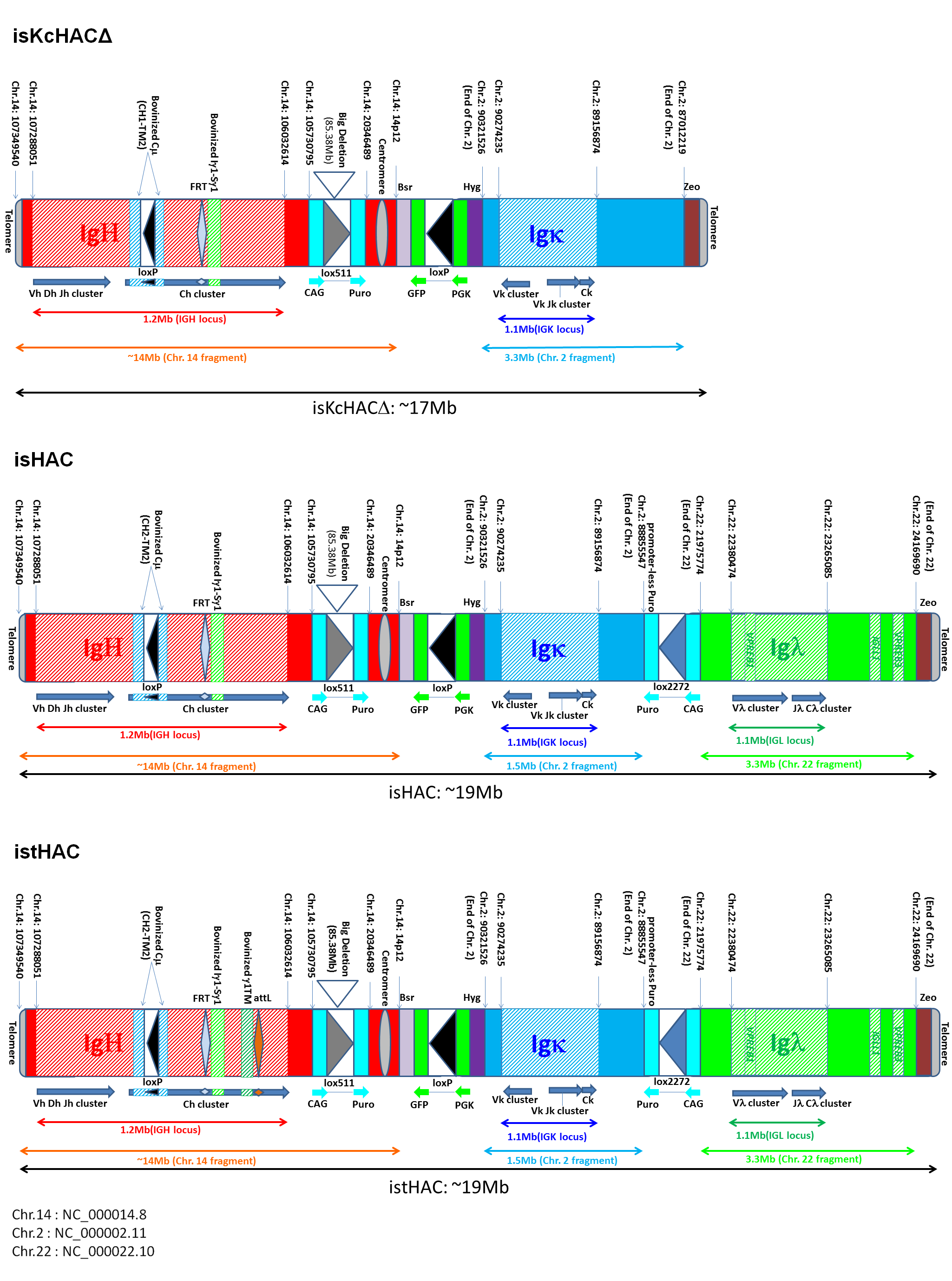

Supplement: S9 Fig — Chr.2, Chr.14 and Chr.22 with the following numbers show the location in the public database sequences from NC_000002.11, for human chromosome 2, NC000014.8 for human chromosome 14 and NC_000022.10 for human chromosome 22, respectively. (TIF) [file pone.0130699.s009.tif]

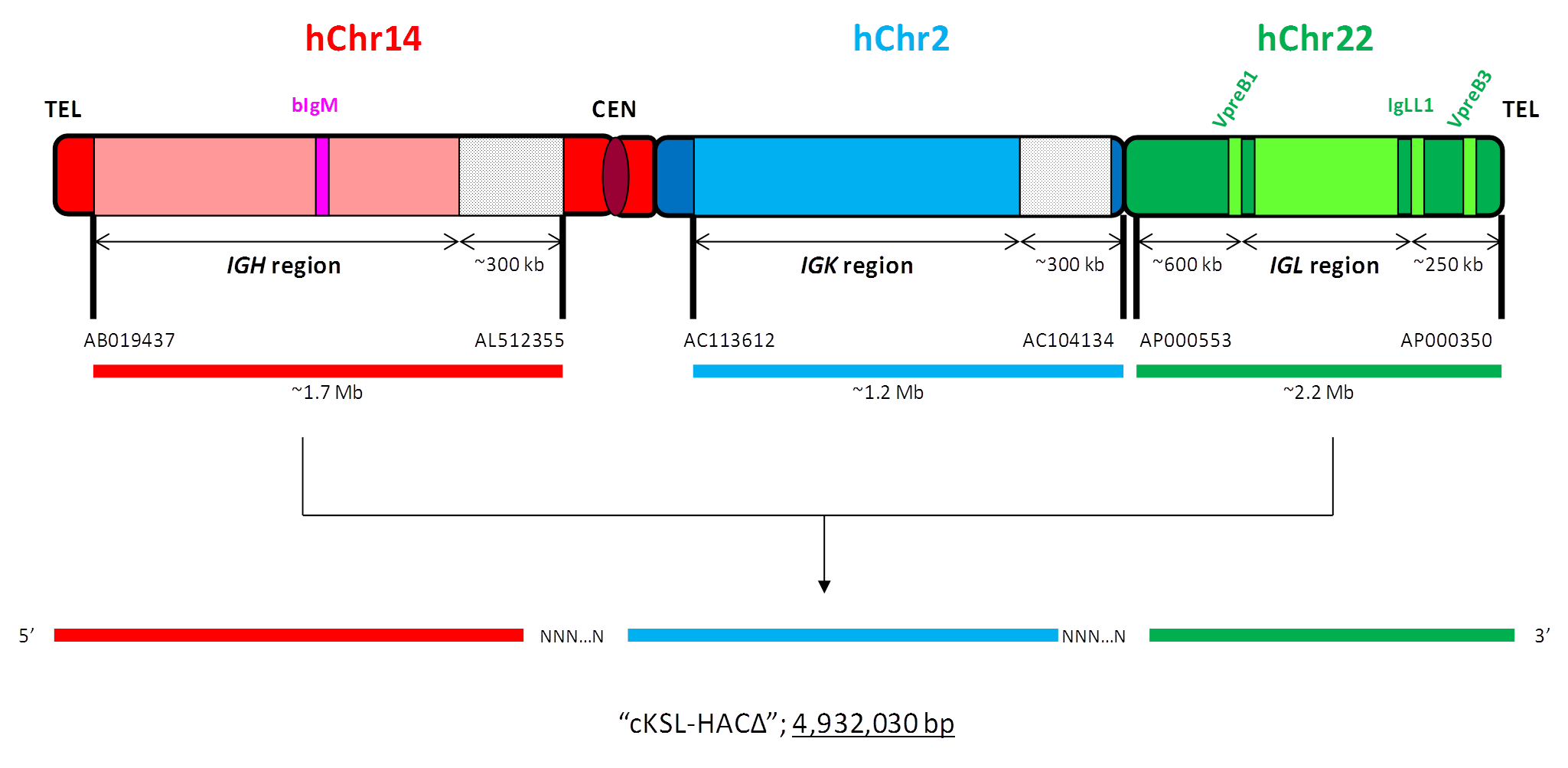

Supplement: S11 Fig — For hChr14, hChr2 and hChr22 fragment sequences, the AB019437 to AL512355, the AC113612 to AC104134, and the AP000553 to AP000350, respectively, were assembled and linked with artificial “NNN…N” to create the 4,932,030 bp DNA sequence as the deduced cKSL-HACΔ vector sequence. (TIF) [file pone.0130699.s011.tif]
