# Supplementary material for: Species-Specific Chromosome Engineering Greatly Improves Fully Human Polyclonal Antibody Production Profile in Cattle
Source: PLoS One. 2015 Jun 24;10(6):e0130699. doi: 10.1371/journal.pone.0130699 (PMC4479556; doi:10.1371/journal.pone.0130699)
Supplement: S1 Table — (DOCX) [file pone.0130699.s012.docx]

**S1 Table.** *p* values for the comparison of serum total hIgG concentrations among different genotypes

|  | A | B | C | D | E | F | G | H |
| --- | --- | --- | --- | --- | --- | --- | --- | --- |
| A |  | 0.2944 | 0.2051 | 0.2897 | 0.9779 | 1.0000 | <0.001* | <0.001* |
| B |  |  | 0.9993 | <0.001* | 0.7643 | 0.1296 | <0.001* | <0.001* |
| C |  |  |  | <0.001* | 0.5705 | 0.1214 | <0.001* | <0.001* |
| D |  |  |  |  | 0.0017* | <0.001* | <0.001* | <0.001* |
| E |  |  |  |  |  | 0.9807 | <0.001* | <0.001* |
| F |  |  |  |  |  |  | <0.001* | <0.001* |
| G |  |  |  |  |  |  |  | 0.4477 |

A, cKSL-HACΔ/TKO (n = 14); B, isHAC/TKO (n = 12); C, istHAC/TKO (n = 13); D, KcHACΔ/TKO (n = 20); E, isKcHACΔ/TKO (n = 17); F, cKSL-HACΔ/DKO (n = 43); G, KcHAC/DKO (n = 25); H, κHAC/DKO (n = 8)

Asterisk (*) shows a significant difference (*p* <0.05).
