# Supplementary material for: Species-Specific Chromosome Engineering Greatly Improves Fully Human Polyclonal Antibody Production Profile in Cattle
Source: PLoS One. 2015 Jun 24;10(6):e0130699. doi: 10.1371/journal.pone.0130699 (PMC4479556; doi:10.1371/journal.pone.0130699)
Supplement: S3 Table — (DOCX) [file pone.0130699.s014.docx]

**S3 Table.** *p* values for the comparison of serum fully hIgG/hIgκ (%)/total hIgG among different genotypes

|  | A | B | C | D | E | F | G | H |
| --- | --- | --- | --- | --- | --- | --- | --- | --- |
| A |  | 0.1039 | 0.0022* | <0.001* | <0.001* | <0.001* | <0.001* | 0.0010* |
| B |  |  | 0.9396 | <0.001* | <0.001* | <0.001* | <0.001* | <0.001* |
| C |  |  |  | <0.001* | <0.001* | <0.001* | <0.001* | <0.001* |
| D |  |  |  |  | 0.9990 | <0.001* | <0.001* | <0.001* |
| E |  |  |  |  |  | <0.001* | <0.001* | <0.001* |
| F |  |  |  |  |  |  | <0.001* | 0.8558 |
| G |  |  |  |  |  |  |  | 0.9998 |

A, cKSL-HACΔ/TKO (n = 13); B, isHAC/TKO (n = 12); C, istHAC/TKO (n = 13); D, KcHACΔ/TKO (n = 19); E, isKcHACΔ/TKO (n = 16); F, cKSL-HACΔ/DKO (n = 42); G, KcHAC/DKO (n = 21); H, κHAC/DKO (n = 3)

Asterisk (*) shows a significant difference (*p* <0.05).
